# Nine Centuries, Seven Words: Cross-Cultural and Cross-Temporal Evidence for the Erosion of Gallo-Lucanian Plant Heritage in Inland Southern Italy

**DOI:** 10.3390/plants15172731

**Published:** 2026-09-07

**Authors:** Stefano De Canio, Ettore Nepote, Shujat Ali, Tahir Shahzad, Renata Sõukand, Naji Sulaiman, Andrea Pieroni

**Affiliations:** 1 University of Gastronomic Sciences, Piazza V. Emanuele II 9, 12042 Pollenzo, Italy; stefanodecanio98@gmail.com (S.D.C.);; 2 Independent Researcher, Tehsil Kabal 19120, Khyber Pakhtunkhwa, Pakistan; alishujat119@gmail.com; 3 Department of Botany, University of Sargodha, Sargodha 40100, Punjab, Pakistan; tahirshahzad174@gmail.com; 4 Biocultural Diversity Lab, Department of Environmental Sciences, Informatics and Statistics, Ca’ Foscari University of Venice, Via Torino 155, 30172 Venezia, Italy; 5 Department of Medical Analysis, Tishk International University, Erbil 44001, Kurdish Autonomous Region, Iraq

**Keywords:** traditional plant knowledge, ethnobotany, cross-cultural comparison, diachronic change, wild food plants, biocultural heritage, Gallo-Lucanian minorities, ethnolinguistics, Basilicata, Southern Italy

## Abstract

This study investigates traditional plant knowledge among Gallo-Lucanian and Lucanian-speaking communities of inland Lucania (Basilicata), Southern Italy, through a cross-cultural and cross-temporal ethnobotanical approach. In a handful of mountain villages, a Gallo-Lucanian substratum survives, carried south by settlers from Piedmont and Liguria between the eleventh and thirteenth centuries; nine hundred years later, we ask what remains of the plants and of the words they brought with them. Fieldwork carried out in 2024 and 2025 with 90 participants in fifteen municipalities, half of them Gallo-Lucanian and half Lucanian speakers, documented 127 identified plant taxa and 12 emic taxa used mainly as wild food and household medicine. The two communities share most of their botanical repertoire: 74 taxa are cited by both, and their Jaccard similarity reaches 0.58. What sets them apart is not the flora but the lexicon, together with the emphasis placed on individual taxa, which differs significantly between the groups. Seven vernacular phytonyms have no counterpart in the surrounding Southern Italian dialects and preserve forms with plausible Piedmontese or Ligurian cognates; several survive attached to plants no longer gathered, so the word outlives the practice it once named. Set against surveys carried out in the same region in 2000–2005 and in inland Liguria, the contemporary repertoire has contracted, particularly among taxa of montane and subalpine distribution, in a landscape reshaped by pastoral decline, land abandonment and recurrent drought. Notably, a substantial share of the gathered taxa is crop wild relatives, among them *Beta vulgaris*, *Daucus carota*, *Lactuca serriola*, *Cynara cardunculus*, *Pastinaca sativa*, *Asparagus acutifolius*, *Diplotaxis tenuifolia*, *Prunus spinosa* and *Pyrus spinosa*, maintained in the interstitial habitats of terraces, field margins and abandoned pastures that these inner areas still preserve. Folk plant uses recorded in minority communities thus provide both a sensitive record of biocultural change and a route to identifying, conserving in situ, and potentially using plant genetic resources in marginal Mediterranean territories.

## 1. Introduction

Recent ethnobotanical scholarship has increasingly challenged static interpretations of local ecological knowledge as a residual cultural inheritance progressively disappearing under modernisation. Instead, attention is increasingly directed to the socio-ecological mechanisms that sustain, restructure, or erode wild food knowledge over time [1,2,3,4,5]. This shift encourages ethnobotanical research to move beyond inventories of plant use towards analyses of knowledge resilience, ecological practice and cultural transmission.

The documentation of folk plant uses among linguistic and cultural minorities occupies a particular position within the study of local ecological knowledge. Where a minority community and a majority community inhabit the same ecological setting, gather in the same woods and pastures and face the same economic pressures, the environment is effectively held constant, and whatever differences emerge in the way plants are named, gathered and used may be referred to cultural inheritance rather than to ecological opportunity. Neighbouring communities sharing a single flora may nonetheless maintain markedly divergent plant repertoires, and such divergences often track linguistic and cultural boundaries more closely than ecological ones [6,7].

Minority communities are also, for the same reason, sentinels of biocultural loss. Minority varieties tend to disappear faster than majority ones [8], and the lexicon that names plants, habitats, seasons and gathering techniques is among the first parts of a language to fall out of use once the practices it describes are abandoned [9]. Examining what remains of that lexicon, and how far it has drifted from the practices it once served, lets us observe biocultural erosion while it is still in progress rather than after it has been completed. The relation is not straightforward: minority-language persistence alone does not guarantee the continuity of foraging knowledge, as recent work among German- and Ladin-speaking Alpine communities has shown [7], and words and practices may erode at quite different rates.

The Gallo-Lucanian settlements of Southern Italy present an unusually deep version of this configuration. Their founders left north-western Italy between the eleventh and thirteenth centuries, and their descendants have lived ever since within a Southern Italian ecological and agrarian world, surrounded by Lucanian-speaking neighbours. Nine centuries is long enough for any purely ecological signal to have been overwritten, and whatever northern element survives in their plant knowledge must have been carried by language, memory and domestic practice rather than by the flora itself. Despite extensive dialectological work on these communities [10,11,12,13], their ethnobiology has never been documented.

Recent studies conducted in northern Italy further demonstrate the complexity and variability of these transformations. Research among German- and Ladin-speaking Alpine minorities showed that minority-language persistence alone does not guarantee the continuity of foraging knowledge when direct interaction with landscapes and gathering practices declines [7]. Conversely, ethnobotanical resilience may persist amid socio-economic transformation when ecological accessibility and the cultural valuation of foraging practices remain active, as demonstrated in post-industrial north-western Italy [14]. Other investigations have documented how wild plant knowledge increasingly undergoes processes of reinvention, heritagisation, gastronomic commercialisation, and adaptive reinterpretation within contemporary food systems and foraging-centred economies [15]. Similarly, research among Middle Eastern diasporic communities in north-western Italy highlighted the central role of ecological adaptation and ingredient availability in reshaping food heritage and ethnobotanical practices after migration [16]. Together, these studies indicate that ethnobotanical change should not be reduced to binary narratives of preservation versus disappearance, but instead interpreted through shifting configurations of ecology, mobility, subsistence practices, and cultural transmission.

This perspective is particularly relevant in Southern Italy, where wild plant gathering historically developed within highly localised agro-pastoral systems characterised by seasonal mobility, marginal agriculture, woodland management, and intensive interaction with synanthropic and ruderal habitats [17]. Basilicata is an especially important case because of its ecological heterogeneity, long-term rural isolation, and remarkable linguistic diversity. Central Lucania (or Basilicata), alongside Lucanian-speaking populations, hosts Gallo-Lucanian communities established by former migrants from north-western Italy between the eleventh and thirteenth centuries and Arbëreshë communities of Albanian origin established during the late medieval period [10,12]. The co-occurrence of linguistic and ecological diversity in such regions is particularly relevant from a biocultural diversity perspective, since linguistic erosion often parallels the weakening of ecological knowledge systems and culturally embedded environmental practices [9]. These populations maintained varying degrees of linguistic distinctiveness while simultaneously adapting to Southern Italian ecological conditions and interacting for centuries through shared agro-pastoral systems and regional exchange networks.

The Gallo-Lucanian settlements are particularly significant from a biocultural perspective because they represent a rare long-term case of migration-associated ethnobotanical adaptation within the Mediterranean Basin. Migration frequently reshapes food knowledge through selective retention of culturally meaningful resources and adaptation to newly available ecologies [18]. Similar processes of selective ethnobotanical adaptation have been documented among long-established minority communities in the Eastern Mediterranean, where migrant groups adopted local foraging repertoires while preserving a limited number of culturally distinctive plant uses and phytonyms linked to ancestral identity [6]. Such evidence suggests that migration-driven ethnobotanical adaptation often produces hybrid knowledge systems rather than simple retention or replacement. While dialectological research has extensively examined the linguistic evolution of these communities [11,13], their ethnobotanical dimensions remain insufficiently explored. Existing ethnobotanical studies in Basilicata have documented the richness of medicinal and wild-food plant traditions [19,20]. Yet, few investigations have quantitatively examined how migration history, ecological restructuring, socio-economic transformation, and minority-language persistence interact diachronically to shape local plant-use repertoires.

At the same time, contemporary rural transformation across Southern Italy is substantially altering the ecological conditions under which ethnobotanical knowledge has historically been reproduced. Land abandonment, declining pastoral mobility, demographic ageing, agricultural marginalisation, and migration towards urban centres are progressively reducing everyday exposure to habitats historically associated with seasonal gathering practices. Such processes affect not only the transmission of practical ecological knowledge but also the ecological salience of species embedded within local food systems, medicinal practices, and vernacular classifications. Ethnobotanical repertoires therefore become selectively reorganised rather than uniformly disappearing, with certain taxa and practices retained, simplified, hybridised, or abandoned according to changing socio-ecological conditions [1,21]).

These dynamics also help explain the relationship between minority identity and everyday ecological practices. Recent research among Arbëreshë communities in Southern Italy demonstrated how culturally distinctive consumption practices may persist as markers of collective identity through embodied domestic and social practices that extend beyond linguistic continuity alone [22]. This perspective suggests that biocultural resilience depends not only upon symbolic cultural retention but also upon sustained material interaction with landscapes, foods, and ecological resources. Because language endangerment is strongly associated with socio-economic integration, urbanisation, and demographic restructuring, minority linguistic systems often face pressures similar to those affecting the transmission of ecological knowledge [8]. Against this background, the present study investigates the cross-cultural dimensions and ethnobotanical knowledge among Lucanians and Gallo-Lucanian speakers in Central Basilicata. Gallo-Lucanians have never been studied in ethnobiology; they are descendants of settlers from NW Italy who migrated to southern Basilicata during the Middle Ages, leaving distinctive linguistic traces that survive to this day [10,12]. Their origins can be traced to the area between southern Piedmont and the Ligurian hinterland, particularly the mountainous province of Savona, a territory long controlled by the Aleramici Marquises of Monferrat [11]. The Gallo-Lucanian resettlement of Lucania forms part of a longer sequence of population movements that began under Lombard rule; however, the communities studied here settled in successive waves between the eleventh and thirteenth centuries. Three political powers drove the process: the Lombards, whose Principality of Salerno (seventh to eleventh centuries) established the political framework; the Normans, who dominated from the eleventh to the twelfth century; and the Angevin rulers, from the thirteenth century onward. Throughout this article we therefore date the settlement to the eleventh–thirteenth centuries, in line with the Abstract. The resettlement was largely strategic; Norman and Angevin lords needed loyal subjects to control newly conquered territories, secure landing areas such as the Gulf of Policastro, and maintain key overland routes connecting Naples to Taranto [23].

Over the centuries, these northern settlers blended with the local population, and their Gallo-Lucanian dialects gradually absorbed southern linguistic elements. Today, small linguistic islands in Basilicata still reflect this medieval migration, representing a remarkable cultural and historical legacy embedded in the dialects of a few villages in southern Italy.

A note on terminology is needed here. Throughout this article the communities studied, their inhabitants and their speech are called “Gallo-Lucanian”, and the surrounding majority group and its dialect “Lucanian”. The Gallo-Lucanian varieties descend from the Romance dialects of north-western Italy (Piedmontese, Ligurian, Lombard and Emilian-Romagnol) carried south by medieval settlers, and it is under that ancestry that they appear in the dialectological literature [10,11,12,13,24]. What survives in Basilicata today is not a separate dialect: after nine centuries of contact it is a substratum within an otherwise Lucanian dialect, audible in intonation, prosody and cadence and in a limited set of lexical items (see Section 2.2).

By integrating contemporary ethnobotanical fieldwork with historical datasets from Southern and North-western Italy, the study aims to: (1) document contemporary medicinal and gastronomic plant uses among linguistically diverse communities in Basilicata; (2) examine how socio-economic transformation, ecological restructuring, and changing forms of landscape interaction have altered ethnobotanical repertoires over time; (3) evaluate the persistence of ethnobotanical and ethnolinguistic elements associated with NW Italy among Gallo-Lucanian communities; and (4) explore the relationship between minority-language persistence, locals’ exposure to nature, and the resilience of local ecological knowledge systems. More broadly, the study contributes to debates on biocultural diversity by examining how ethnobotanical knowledge is shaped through interactions among ecology, subsistence practices, and socio-economic change.

## 2. Materials and Methods

### 2.1. Study Area

**Figure 1:**
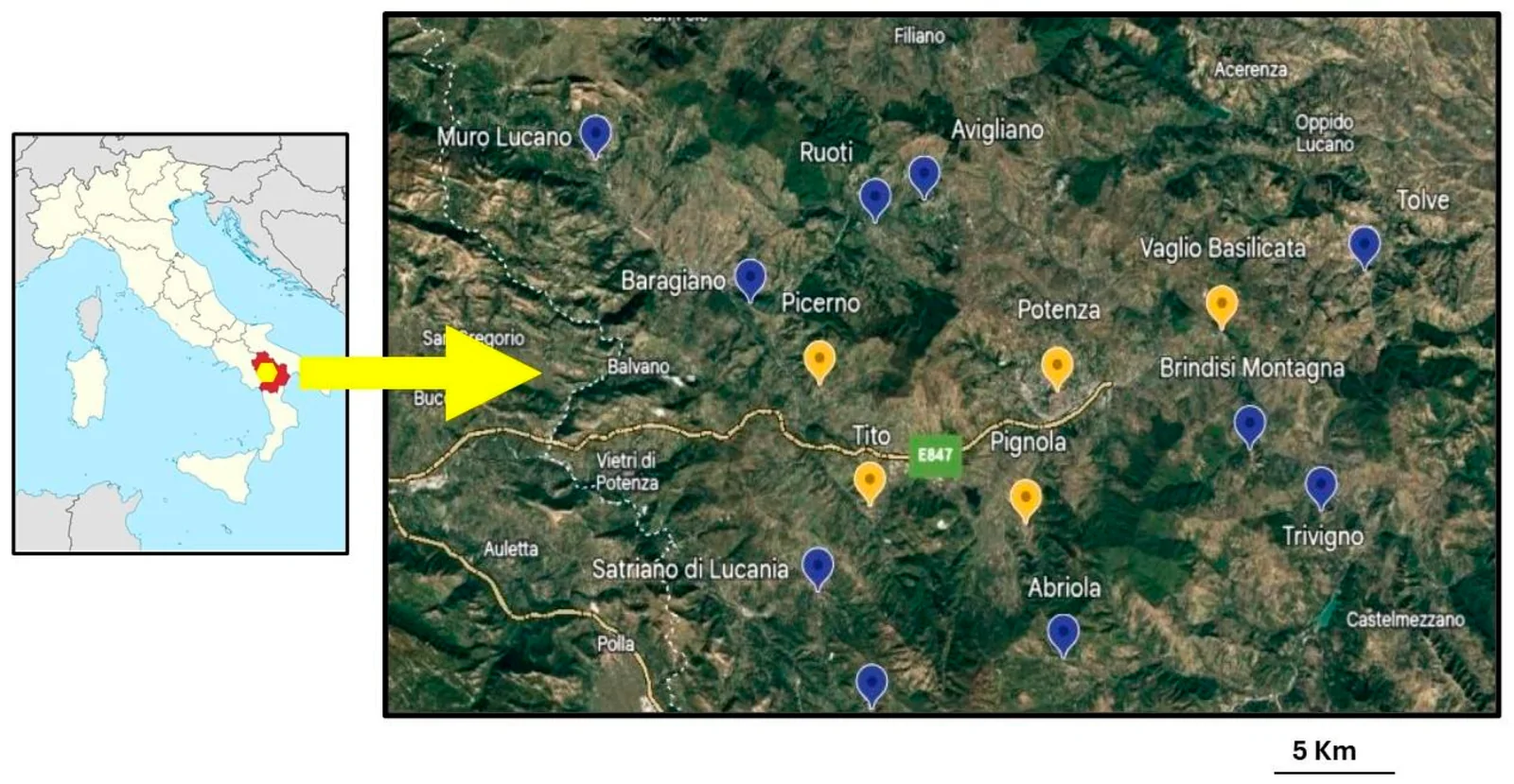
Lucania (Basilicata) in red within Italy and map of the (yellow) study area in Central Lucania-the villages in yellow retain a Gallo-Lucanian dialect, while the surrounding villages in blue speak an autochthonous Lucanian dialect.

The study was conducted in Basilicata (Lucania), Southern Italy, primarily in the province of Potenza (Figure 1). Fieldwork covered 15 municipalities across mountainous inland areas with elevations ranging from approximately 500 to 1200 m a.s.l. The study area includes both Gallo-Lucanian-speaking settlements and neighbouring Lucanian-speaking communities.

The Gallo-Lucanian municipalities included: Picerno (5590 inhabitants, 721 m a.s.l.), Pignola (6841 inhabitants, 926 m a.s.l.), Potenza (63,661 inhabitants, 819 m a.s.l.), Tito (6983 inhabitants, 650 m a.s.l.), and Vaglio Basilicata (1795 inhabitants, 954 m a.s.l.). Surrounding Lucanian municipalities were: Abriola (1206 inhabitants, 957 m a.s.l.), Avigliano (10,435 inhabitants, 827 m a.s.l.), Baragiano (2469 inhabitants, 624 m a.s.l.), Brindisi Montagna (768 inhabitants, 800 m a.s.l.), Muro Lucano (4877 inhabitants, 600 m a.s.l.), Ruoti (3187 inhabitants, 756 m a.s.l.), Sasso di Castalda (719 inhabitants, 949 m a.s.l.), Satriano di Lucania (2236 inhabitants, 650 m a.s.l.), Tolve (2881 inhabitants, 568 m a.s.l.), and Trivigno (602 inhabitants, 735 m a.s.l.)

**Figure 2:**
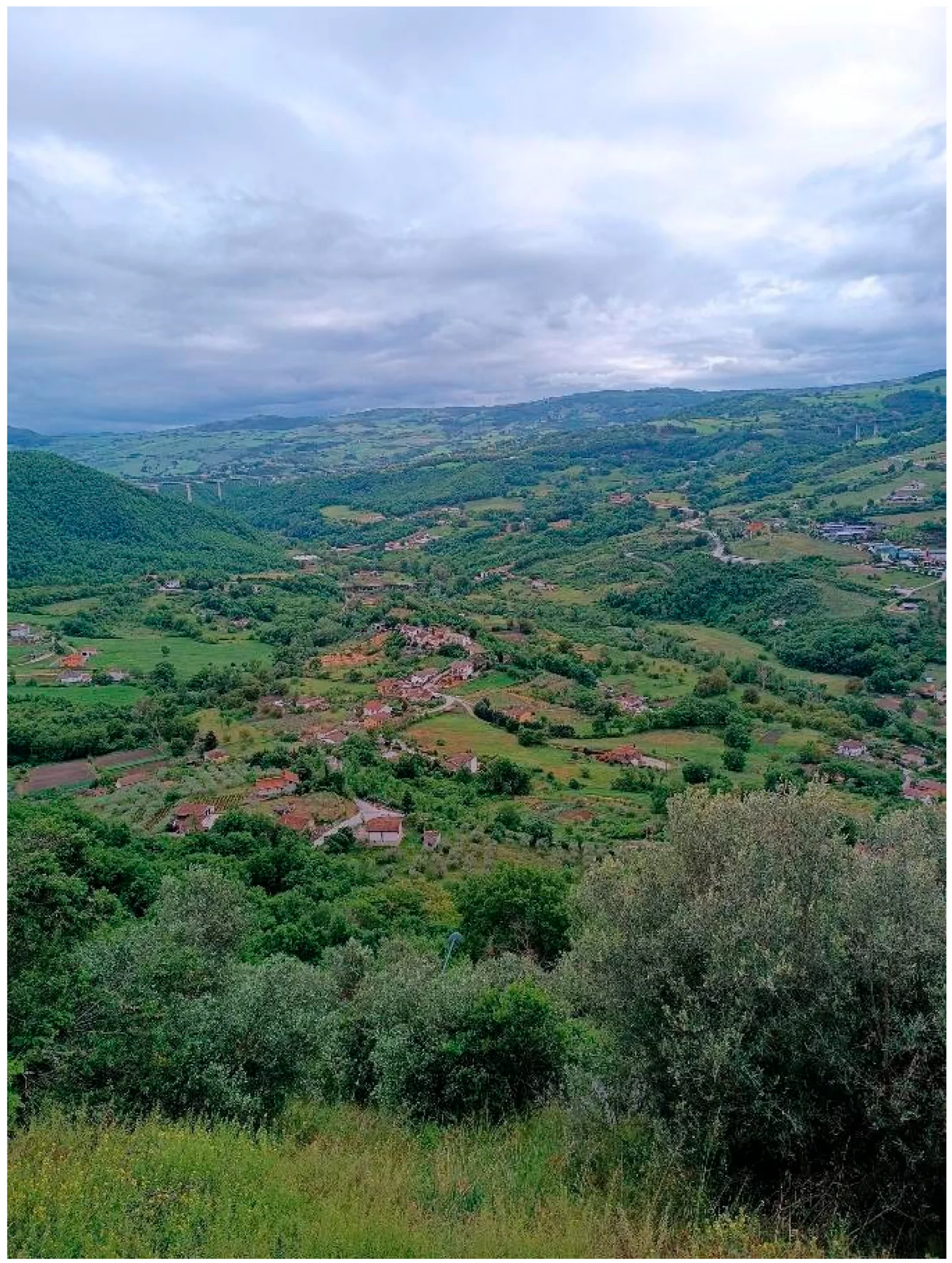
Spring landscapes in the Gallo-Lucanian settlements (Photos: S. De Canio).

The investigated territory belongs to the southern Apennine system and is characterised by heterogeneous ecological conditions including mixed deciduous woodlands, ruderal habitats, agro-pastoral landscapes, Mediterranean shrublands, and mountain grasslands. Historically, these environments supported subsistence agriculture, transhumant pastoralism, and extensive wild plant gathering (Figure 2).

The fifteen municipalities form a single compact block of approximately 815 km^2^ centred on the Basento valley, with maximum diagonals of 46 and 38 km and elevations ranging from 332 to 1722 m a.s.l. (median 810 m). Gallo-Lucanian and Lucanian villages are interdigitated within this block rather than occupying separate districts, so the two groups of participants draw on the same regional flora and, in most cases, on directly adjacent territory. This matters for interpreting the between-group comparison in Section 3.5.

The region has experienced substantial socio-economic transformation during the twentieth and twenty-first centuries, including rural depopulation, decline of traditional pastoralism, agricultural abandonment, and demographic ageing. These processes have coincided with increasing ecological succession and landscape restructuring.

Historically, multiple migration processes and linguistic interactions have also shaped the area. Gallo-Lucanian communities descend from settlers originating from north-western Italy between the eleventh and thirteenth centuries. In contrast, Arbëreshë communities derive from Albanian migrations during the late medieval and early modern periods.

### 2.2. Ethnobotanical Data Collection

We conducted fieldwork between August 2024 and July 2025 through semi-structured interviews, participant observation, and informal focus-group discussions. Researchers interviewed 90 participants across the municipalities investigated, with equal representation of Gallo-Lucanian- and Lucanian-speaking communities (N = 45 interviews in each group).

We selected interviewees through snowball sampling and opportunistic encounters in villages, agricultural areas, forests, and pastoral environments. We prioritised individuals recognised locally as holders of traditional ecological knowledge, especially elderly farmers, shepherds, gatherers, and those involved in traditional food preparation [25].

Selection followed explicit inclusion and exclusion criteria. Participants had to be long-term residents of one of the fifteen study municipalities and be recognised locally as holders of traditional plant knowledge; preference was given to elderly farmers, shepherds, gatherers and people involved in traditional food preparation, and a smaller number of younger participants were approached where their knowledge derived directly from a grandparent who had lived under pre-globalisation conditions. Individuals whose knowledge the interviewer judged to have been substantially displaced by prolonged residence outside the area, through migration, employment or education, were not interviewed further. Recruitment combined opportunistic encounters in villages, on agricultural land, in pastures and in woodland with snowball sampling through local social networks, and interviews took place at field margins and gathering sites, in village streets and in participants’ homes. Table 1 summarises the sampling frame and the participant characteristics recorded during the fieldwork.

Assigning participants to the Gallo-Lucanian or Lucanian group requires a clarification we consider important enough to state before the criterion itself, because it qualifies much of what follows. Today, no discrete Gallo-Lucanian dialect stands apart from the surrounding Lucanian one, and we do not claim that there is. What our participants speak in the five Gallo-Lucanian villages is a Lucanian dialect within which a Gallo-Lucanian substratum is audible as intonation and prosody, as a characteristic sing-song cadence (locally described as a *cantilena*), and as a scattering of individual lexical items, several of which are the phytonyms discussed in Section 3.6. It is not a separate linguistic system with its own speakers, and no participant could be classified by asking which variety they spoke.

Assignment was therefore made on residence in a municipality independently documented as Gallo-Lucanian in the dialectological literature, not on self-declared speech or ethnicity. Participants resident in Picerno, Pignola, Potenza, Tito and Vaglio Basilicata—identified as Gallo-Lucanian by Rohlfs [10] and confirmed by the recent linguistic atlas of Basilicata [24]—were classified as Gallo-Lucanian; participants resident in the ten surrounding municipalities were classified as Lucanian. All participants were lifelong residents of their municipality, which is one of the inclusion criteria in Table 1, so assignment is unambiguous at the individual level. It nonetheless remains a geographical and dialectological criterion, not a genealogical or strictly linguistic one: religion and language long ago ceased to constitute barriers between the two groups, considerable intermarriage and movement have occurred between them, and the two populations are neither genetically nor socially discrete. The comparison reported below is therefore between the inhabitants of villages with different documented linguistic histories, not between two speech communities.

We noted gender, approximate age, principal occupation, educational level, and length of residence for every participant and report them above, along with length of gathering experience, which was lifelong in every case. All participants reported gathering wild plants since childhood, so the variable does not discriminate within this sample. Two features of the fieldwork account for the omission. First, the protocol was use-centred rather than participant-centred: the recording unit was plant use, and the interview schedule focused on gathering, preparation and nomenclature rather than the interviewee. Second, a substantial proportion of the interviews were unplanned encounters in fields, pastures and village streets with elderly people who had agreed to talk about plants; administering a personal-data form in that setting would have been intrusive and, in our judgement, would have reduced participation. The verbal consent obtained covered the ethnobotanical material, and we did not consider ourselves entitled to record and archive personal characteristics beyond those participants volunteered.

This is a genuine limitation and we do not minimise it. Two considerations mitigate it, though do not remove it. The inclusion and exclusion criteria in Table 1 constrain the sample on precisely the dimensions that the missing variables would describe: every participant was a long-term resident of a study municipality, every one was selected as a recognised holder of traditional plant knowledge, and individuals whose knowledge had been displaced by prolonged absence were excluded. The sample is therefore homogeneous by construction rather than by chance, and a mean age of 70 years places it unambiguously in the generation that still practised agro-pastoral subsistence. We note that both subsamples contain more men than women, which may bias the recorded repertoire towards plants associated with pastoral and field work rather than with domestic preparation. The sample cannot claim representativeness of the general population of the study area, and Section 4 states this as the first limitation of the study.

We cannot retrospectively reconstruct the missing variables: the field notes and audio archive were organised by plant and use rather than by participant, and re-contacting all ninety participants two years after the fieldwork, in a population of this age, is not realistic. We therefore report what exists, and we treat systematic socio-demographic recording as a design requirement for the follow-up surveys proposed in Section 4.

Interviews explored wild food plant gathering; medicinal plant uses; vernacular plant nomenclature; habitat associations; temporal changes in plant use; perceptions of environmental and social transformation; and the intergenerational transmission of knowledge. We collected audio recordings, field notes, and photographic documentation with prior informed consent. The Ethics Committee (Comitato Etico) of the University of Gastronomic Sciences of Pollenzo reviewed and approved the study at meeting No. 4/2024 on 17 June 2024, and all fieldwork was carried out after that date. Before each interview, participants were informed of the study’s aims, the intended use and storage of the data, and their right to decline any question or withdraw at any point; verbal informed consent was obtained and recorded in the field notes. The research followed the Code of Ethics of the International Society of Ethnobiology [26], including its provisions on prior informed consent, participant confidentiality, and the return of results to the communities concerned.

Plant taxa were identified using the Flora d’Italia ([27]–2019). We collected voucher specimens whenever the plant was available at a suitable phenological stage during the interview or the accompanying field walk, and deposited them in the Herbarium of the University of Gastronomic Sciences of Pollenzo. Because many interviews took place outside the gathering season, and because several taxa were discussed only as memory-based practices, we could not collect vouchers for every recorded taxon. Of the 127 identified taxa listed in Table 3, 21 are documented by vouchers collected during the present fieldwork (UNISGLU codes) and a further 15 by vouchers collected during our earlier surveys in Basilicata and Calabria (CALARB codes), which are cited here as comparative material and do not originate from the present study; considering wild taxa alone, 15 of the 97 species recorded are backed by a voucher collected during this fieldwork. For the remaining taxa, identification was based on the illustrated keys of the Flora d’Italia together with direct observation of the plant in the field or of photographs taken during the interview. This procedure is considered reliable for common trees, shrubs and unmistakable herbs, and for cultivated species. For sixteen taxa, however, no plant could be observed, and identification rests solely on the vernacular name and the morphological and ecological description supplied by the participant; these are marked with an asterisk, and their species-level attribution should be treated as provisional. Plants that could not be attributed with reasonable confidence even at genus level are reported as unidentified emic taxa. A small number of entries are deliberately reported at genus level or as species aggregates (for example *Allium* spp., *Valerianella* sp., *Rumex crispus* and allied species, and Sinapis arvensis together with *Hirschfeldia incana* and *Rapistrum rugosum*) because participants themselves did not distinguish the constituent species; these are not counted among the sixteen provisional identifications.

To describe the elevational range of the harvested taxa, each species was assigned to the highest elevational belt at which it is reported to occur in the Flora d’Italia ([27]–2019), and species were grouped into five belts representative of the study area: lowland (0–200 m a.s.l.), hilly (200–1000 m a.s.l.), mountain (1000–1400 m a.s.l.), subalpine (1400–1800 m a.s.l.), and alpine (1800–2700 m a.s.l.). This variable describes the potential upper elevational range of each taxon, not the elevation at which participants actually gathered it: a widely distributed species is assigned to a high belt even when it was collected beside the village. Gathering elevations were not recorded systematically in either the contemporary or the historical surveys, so the analysis reported below compares the floristic range limits of the harvested repertoires and is interpreted only in those terms.

For the historical datasets, which likewise lack complete botanical vouchers, taxonomic validation followed a multi-step protocol based on voucher-confirmed identifications where available, comparison of vernacular names, ecological plausibility, habitat correspondence, the historical ethnobotanical literature and regional floristic occurrence. Uncertain taxa and ecologically implausible identifications were excluded from the comparative analyses.

### 2.3. Data Analysis

The contemporary ethnobotanical dataset was systematically compared with previously published ethnobotanical investigations conducted by our group in Lucania between 2000 and 2005 among Lucanian and Arbëreshë communities (Arbëreshë: [28], 2002b; Lucanians: [29,30]), and with a survey carried out in 2013 in the North-Western Ligurian Alps by a research group of the University of Genoa [31], not far from the area from which the Gallo-Lucanian communities are believed to have originated.

The comparative framework was designed to identify both diachronic transformations and cross-cultural continuities in local ecological knowledge related to wild food and medicinal plants. Rather than limiting the analysis to simple floristic overlap, the comparison incorporated multiple analytical dimensions, including presence and absence of taxa, culinary and medicinal applications, vernacular nomenclature, ecological associations, and patterns of cultural salience.

Table 2 sets out, for every source entering the comparison, the information needed to judge its comparability with the present survey. Three features of that table govern how to read the diachronic results below. First, each of the two historical repertoires was assembled by merging two earlier publications—one documenting food plants, the other medicinal plants—so the merged repertoire rests on two distinct participant samples recruited at different times. Second, the sampling designs differ from ours in kind and not only in size: the historical surveys selected elderly knowledge holders within one or three villages, whereas the present survey covers fifteen municipalities, and the North-Western Italian survey selected key informants defined by occupation. Third, the range of use categories differs: Cornara et al. [31] recorded thirteen use categories, Pieroni et al. [28] and Pieroni et al. [29] recorded food uses only, and Pieroni et al. [32] and Pieroni and Quave [30] recorded medicinal uses only, whereas we recorded food and medicinal uses together. The cross-temporal comparison therefore compares published repertoires assembled under different designs, not equivalently sampled populations, and we treat it as descriptive throughout.

**Table 2:** Metadata of the sources entering the comparative analysis and the Detrended Correspondence Analysis in Figure 5, compiled from the original publications. N = number of participants. The five DCA repertoires are formed by the seven sources listed: the Arbëreshë repertoire merges Pieroni et al. [28] and Pieroni et al. [32], and the historical Lucanian repertoire merges Pieroni et al. [29] with the Castelmezzano component of Pieroni and Quave [30].

DCA Repertoire	Location/Community	Field-Work	N	Sampling	Interview Method	Plant-Use Scope	Reference
Gallo-Lucanian 2024–2025	Five Gallo-Lucanian municipalities, prov. Potenza	2024–2025	45	Purposive; snowball and opportunistic	Semi-structured; participant observation	Wild food and medicinal	Present study
Lucanian 2024–2025	Ten Lucanian-speaking municipalities, prov. Potenza	2024–2025	45	Purposive; snowball and opportunistic	Semi-structured; participant observation	Wild food and medicinal	Present study
Arbëreshë (food component)	Ginestra, Maschito and Barile, Vulture area, prov. Potenza	2000–2001	68 (62 aged > 50)	Purposive; elderly knowledge holders	Semi-structured and structured; participant observation; group interviews	Wild food plants (*liakra*)	[28]
Arbëreshë (medicinal component)	Arbëreshë villages, northern Basilicata	2000–2001	44 (34 F, 10 M; aged 47–94)	Purposive; elderly knowledge holders	Semi-structured and structured; participant observation	Medicinal plants and other natural remedies	[32]
Lucanian (food component)	Castelmezzano (850 inhabitants), Dolomiti Lucane, central Lucania	2002–2003	86 (elderly, mostly women)	Purposive; elderly knowledge holders	Consented interviews; participant observation; round-table focus groups	Non-cultivated and semi-cultivated food plants and mushrooms	[29]
Lucanian and Arbëreshë (medicinal component)	Ginestra/Zhurë and Castelmezzano	2000–2003	c. 65 per community	Largely random, from the elderly population	Semi-structured; participant observation	Household remedies; ritual healing practices	[30]
North-Western Italian	Upper Tanarello and Arroscia Valleys, Ligurian Alps	2009–2011	65	Purposive; key informants	Semi-structured, open	Thirteen use categories, chiefly medicinal and food	[31]

Constructing the comparative dataset required standardising botanical nomenclature and harmonising use categories across studies with different methodological approaches and sampling intensities. We updated scientific names in accordance with contemporary taxonomic standards to ensure comparability between historical and recent datasets. We retained vernacular phytonyms in their original linguistic forms whenever possible because local plant nomenclature is an important component of biocultural knowledge and reflects historical relationships between language, ecology, and subsistence practices. We paid particular attention to taxa associated with pastoral mobility, forest-edge gathering, seasonal food practices, and medicinal preparations embedded within domestic agro-pastoral economies.

The analytical comparison was not intended to establish strict quantitative equivalence between datasets collected under different temporal and methodological conditions. Instead, the objective was to identify broader socio-ecological trends related to persistence, transformation, diversification, and erosion of ethnobotanical knowledge systems. Diachronic comparison therefore focused primarily on changes in culturally salient taxa, shifts in ecological composition, and modifications in the relationship between local communities and their surrounding environments.

To support ecological interpretation, we assigned each recorded taxon to ecological categories based on habitat affinity and ecological behaviour, using regional floristic databases, ecological surveys, and the Mediterranean vegetation literature. We classified species into broad ecological groups, including synanthropic species, ruderal taxa, agro-pastoral plants, woodland species, grassland taxa, and edge or transitional habitat species. This classification enabled us to investigate whether socio-economic and land-use transformations corresponded to changes in the ecological structure of ethnobotanical repertoires. We placed particular emphasis on taxa historically associated with anthropogenic disturbance regimes, transhumant pastoral systems, hay meadows, cultivated field margins, and mixed agro-silvo-pastoral landscapes, since these ecological settings have undergone substantial transformation in recent decades.

The statistical analysis combined descriptive ethnobotanical approaches with multivariate exploratory techniques to examine relationships among temporal, ecological and cultural variables. We constructed presence-absence matrices and citation frequencies for all datasets and analysed them using Detrended Correspondence Analysis (DCA). To test whether the two contemporary communities differ more than expected by chance, the taxon × community matrix of citation counts was analysed by a chi-square test of independence, restricted to the 65 taxa with at least five citations so that expected-frequency requirements were met; the Jaccard index additionally quantified the overlap between the two repertoires. Because the historical datasets were collected under different sampling designs, we did not apply an inferential test across the diachronic comparison; we present the results descriptively.

We also calculated comparative similarity indices and ecological frequency distributions to assess shifts in plant repertoire across time and between cultural groups. These analyses focused especially on the persistence of northern and mountain-associated ethnobotanical elements, the decline of taxa linked to traditional agro-pastoral systems, and the restructuring of culturally salient species amid socio-economic transformation and ecological change. Descriptive statistical analyses also evaluated variation in ecological categories, medicinal and culinary applications, and patterns of knowledge concentration among the investigated communities.

Taken together, the comparative and statistical framework adopted in this study allowed ethnobotanical change to be interpreted not merely as the disappearance of isolated plant uses, but as a broader restructuring of socio-ecological relationships shaped by changing forms of landscape interaction, labour organisation, subsistence practices, and intergenerational knowledge transmission.

### 2.4. Quantitative Ethnobotanical Indices

Following the reviewers’ recommendation, we also analysed the contemporary dataset using standard quantitative ethnobotanical indices, computed separately for each community and for the pooled sample (N = 90; N = 45 per group). A use report (UR) is one plant–use combination reported by one participant. The Use Value of a taxon (UV = ΣU_i_/N) and the Cultural Importance Index (CI = Σ_u_Σ_i_UR_u__i_/N) were calculated as defined by Tardío and Pardo-de-Santayana [33]; at the level of the single taxon the two coincide in our dataset, and we report CI. The Relative Frequency of Citation (RFC = FC/N) follows Tardío and Pardo-de-Santayana [33], where FC is the number of participants who cited the taxon at least once. The Informant Consensus Factor (ICF = (N_ur_ − N_t_)/(N_ur_ − 1)) was computed per use category following Trotter and Logan [34], where N_ur_ is the number of use reports in the category and N_t_ the number of taxa contributing to it. We assigned use reports spanning more than one ailment to the first ailment the participant named.

One qualification applies to RFC, and we state it in full because it determines how to read the column. FC requires counting each participant once per taxon, which presupposes a participant × taxon matrix. The fieldwork did not generate one: the recording unit was plant use throughout, and the field notes and audio archive were organised by plant and use rather than by interviewee, so we cannot retrospectively recover the number of distinct participants behind a given taxon. We therefore report RFC as a lower bound, taking FC as the largest number of citations recorded for any single use of the taxon, since each participant can contribute at most one citation to a single use. Two consequences follow. Where a taxon is used in several distinct ways, the bound is loose, and the true RFC is higher than the value tabulated. Where a row of Table 3 groups more than one preparation or ailment under a single entry, the bound is inflated; values are accordingly capped at 1.00. CI, UV and ICF are unaffected, since all three are defined on use reports rather than on participants. We report RFC in this bounded form rather than omitting it, because the ranking it produces is informative even where the absolute values are not. Still, do not compare these figures with RFC values from participant-based surveys.

The Detrended Correspondence Analysis requires a separate note, because it used different material than the indices above. The ordination was computed on a genus-level presence/absence matrix of 225 genera × five datasets, in which each cell records only whether the genus appears in that dataset’s recorded repertoire (1) or does not (0); citation frequencies were not used. The five datasets, with the number of genera recorded in each, are: Gallo-Lucanian 2024–2025 (95 genera), Lucanian 2024–2025 (86), Arbëreshë of Lucania 2000–2001 (110), Lucanian 2002–2003 (122) and North-Western Italian 2009–2011 (144); their provenance is given in Table 2. The genus was adopted as the operational taxonomic unit because a substantial proportion of the taxa in the historical and external datasets are reported only to genus, or as species aggregates that cannot be resolved against our own material; aggregating our species-level records to genus was the only way to place all five repertoires on a common footing. The matrix further excludes genera used only as cultivated food plants. This restriction was necessary because the source studies are not congruent in scope: some documented non-cultivated plants only, whereas others covered cultivated species as well, so that retaining cultivated taxa would have made the apparent richness of a repertoire a function of the scope of the original study rather than of the gathering practices it recorded. The exclusion applies only to cultivated food plants. Medicinal uses were retained whether the plant is gathered or grown, because the medicinal surveys among the sources documented cultivated as well as wild species, so removing cultivated medicinal taxa would have truncated those repertoires asymmetrically; the food surveys, by contrast, recorded non-cultivated plants only, so cultivated food taxa are not comparable across sources and were dropped. The criterion was applied source by source rather than to the genus as a whole, since the same genus may be gathered in one community and grown in another: *Armoracia rusticana*, for example, is “tolerated” rather than cultivated at Castelmezzano [29] and is retained for that dataset alone, being a cultivated food plant elsewhere. Three consequences follow and are carried through the interpretation in Section 3.7: the ordination describes compositional overlap between repertoires and not their cultural salience, so a genus recorded once weighs exactly as much as one recorded forty times; it operates at a taxonomic resolution coarser than the rest of the analysis, so it cannot detect species-level substitutions within a genus; and it describes the gathered, non-cultivated component of each repertoire only, so it is not comparable with the taxon counts reported elsewhere in this article, which include cultivated species. We provide the full matrix as Supplementary Material.

We computed indices in Python 3.11 (pandas, SciPy). The chi-square test of independence reported in Section 3.5 was performed on the taxon × community matrix of citation counts, restricted to taxa with at least five citations so that expected-frequency requirements were approximately met; the Jaccard index was computed on the presence–absence of taxa in the two repertoires.

## 3. Results and Discussion

### 3.1. The Ethnobotanical Heritage: Composition of the Repertoire

Across all investigated communities, we documented 127 identified taxa (a small number of entries comprising two or more closely related species that informants did not distinguish) and 12 emic taxa that could not be identified botanically. The presence of folk categories that do not map onto single Linnaean species is expected rather than anomalous: folk classifications group organisms according to perceptual salience and practical use, so that a single vernacular name may cover several botanical species that behave identically in the kitchen [35,36]. Of the 127 identified taxa, 74 were cited by both communities, 30 exclusively by Gallo-Lucanian and 23 exclusively by Lucanian participants, so that the Jaccard similarity of the two repertoires is 74/127 = 0.58. The predominance of wild taxa, particularly within Asteraceae, Rosaceae, Lamiaceae, Apiaceae and Poaceae, confirms the centrality of spontaneous vegetation within local food systems and household medicine [37]. Wild greens collected from ruderal habitats, field margins, woodland edges and disturbed agro-pastoral environments constituted one of the most recurrent categories within the dataset. Species such as *Cichorium intybus*, *Sonchus oleraceus*, *Foeniculum vulgare*, *Malva sylvestris*, *Borago officinalis*, *Taraxacum officinale* and Urtica dioica displayed high citation frequencies among both Gallo-Lucanian and Lucanian informants, suggesting the persistence of a shared ethnobotanical substratum that transcends linguistic boundaries [38].

At the same time, the data also reveal culturally specific plant repertoires associated with Gallo-Lucanian communities. Several taxa marked in the dataset as cultural indicators show strong correspondence with ethnobotanical traditions documented in Piedmont and Liguria, while being absent or marginal in neighbouring Lucanian-speaking areas. These taxa include *Allium ursinum*, *Equisetum arvense*, *Humulus lupulus*, *Valeriana officinalis*, *Nasturtium officinale*, and Pastinaca sativa. Their persistence suggests that certain ethnobotanical practices serve as biocultural markers of historical migration and long-term cultural memory.

Importantly, these findings support the interpretation that ethnobotanical knowledge should not be approached solely as a utilitarian adaptation to local ecology. The selective preservation of specific northern-associated taxa indicates that plant knowledge may also operate as a repository of cultural identity and historical continuity. In this sense, the botanical repertoire reflects not only ecological adaptation to Southern Italian environments but also the persistence of mnemonic and linguistic structures associated with the medieval Gallo-Lucanian colonisation of Basilicata. This aligns with the concept of biocultural memory, whereby ecological knowledge persists as an embodied archive of historical experience, migration, and identity even under conditions of socio-economic transformation [39].

**Table 3:** Wild food and medicinal plants recorded in Central Lucania during the current fieldwork, along with their local names and uses. Bold folk names indicate taxa quoted by non-Southern Italian terms. An asterisk (*) marks the sixteen taxa for which no voucher specimen was collected and whose identification at species level rests on the vernacular name and on the description given by participants, rather than on examination of the plant; these identifications are provisional and are discussed in Section 4.

Botanical Taxon/Taxa, Botanical Family, Status, and Voucher Code(s)	Vernacular Name(s)	Part(s) Used	Recorded Local Preparation(s) and Use(s) F: Wild Food; M: Medicinal	Number of Quotes Among Gallo-Lucanians (G) and Lucanians (L)
*Achillea millefolium* L. Asteraceae (wild)	*Millefiori* L	Flowering tops	M: Tea for kidney problems	5 L
*Allium cepa* L. Amaryllidaceae (cultivated)	*Cëpoddë* L	Bulbs	M: Raw, rubbed on the skin for burns	1 L
			M: Decocted in milk against cough	1 L
*Allium sativum* L. Amaryllidaceae (cultivated)	*Aglë* GL	Bulbs	M: Raw, rubbed on the skin or consumed as a vermifuge	9 G 9 L
			M: Crushed, mixed with vinegar and mint, macerated in water and breathed as a vermifuge	5 L
			M: Decoction, drunk for diabetes and blood pressure	1 G 3 L
			M: Raw, topically applied for toothaches	2 L
			M: Crushed, decocted and drunk as a vermifuge	1 L
			M: Consumed raw for stomachaches	1 G
			M: Raw in a poultice, externally applied for muscular pains	1 G
*Allium schoenoprasum* L. Amaryllidaceae (wild) *	*Erba Cipollina* L.	Leaves	F: Raw in salads	2 L
*Allium* spp. Amaryllidaceae (wild)	*Aglë Ardiettë* G *Aglë Salvaggë* G	Whole plant	F: Raw or cooked as a condiment	2 G
*Amaranthus retroflexus* L. Amaranthaceae (wild)	*Iëtillë* L	Young leaves	F: Boiled	2 L
*Anacyclus clavatus* (Desf.) Pers. Asteraceae (wild) and Anthemis cotula L. Asteraceae (wild) UNISGLU011	*Puppëma* GL *Puppëta* GL	Leaves	F: Recreational tea M: Decoction as a vermifuge	1 L 1 G 1 L
*Armoracia rusticana* G.Gaertn., B.Mey. & Scherb. Brassicaceae (cultivated)	*Rafanë* GL	Roots	F: Raw, grated on pasta and in omelettes	17 G 13 L
*Arundo donax* L. Poaceae (wild)	*Cannë* G	Nodal diaphragm membranes	M: Posed on the wound as a haemostatic	4 G
*Asparagus acutifolius* L. Asparagaceae (wild) CALARB08	*Spalëcë* GL	Shoots	F: Boiled and fried in omelettes	24 G 23 L
*Asphodeline lutea* Rchb. Asphodelaceae (wild)	*Mazza Hattë* L	Young stems	F: Roasted	4 L
*Asplenium ceterach* L. Aspleniaceae (wild)	*Cëassa Vuojë* G *Spaccaprete* G	Leaves	M: Infusion for the kidneys	4 G
*Atropa belladonna* L. Solanaceae (wild)	*Belladonna*	Not specified	M: abortive	1 G
*Avena sativa* L. Poaceae (cultivated)	*Avenë* L *Biama* L *Biava* GL	Fruits	M: Decoction for stomachaches or as a milk substitute for children	17 G 13 L
			F: Crushed to make flour for pasta	1 G
*Beta vulgaris* L. Amaranthaceae (wild) CALARB03	*Bietë* **G** *Ierë* L	Leaves	F: boiled	11 G 6 L
*Borago officinalis* L. Boraginaceae (wild) UNISGLU029 UNISGLU020	*Burrain* GL *Vurrascën* GL *Vërrainë* G *Suha Suha* G	Young leaves	F: Boiled	26 G 21 L
		Leaves	M: Decoction for stomachache	6 G 1 L
		Leaves and flowers	M: Decoction as a galactagogue	3 G 1 L
		Stem	F: Boiled	1 L
		Flowers	F: Raw sucked as candies	1 G
*Brassica oleracea* L. Brassicaceae (cultivated)	*Caulë* L	Leaves	M: Heated, greased with oil and placed on the belly for digestive troubles	2 L
		Stem	M: Raw, rubbed on the anus as a laxative	2 L
*Carduus pycnocephalus* L. Asteraceae (wild) and possibly other Asteraceae UNISGLU025	*Cardonë* G *Quardonë* GL	Very young leaves	F: boiled	2 G
*Castanea sativa* Mill. Fagaceae (semi-domesticated)	*Castagna* GL	Fruits	F: Dried and crushed as flour	1 L
			M: Dried and decocted for cold	1 G
*Centaurium erythraea* Rafn. Gentianaceae (wild)	*Erba Dë La Frevaë* L	Leaves	M: Decoction for fever	1 L
*Chenopodium album* L. Amaranthaceae (wild) CALARB21	*Spinacë* L	Leaves	F: Boiled	2 L
*Cnicus benedictus* L. Asteraceae (wild) *	*Cardo Santo* L	Leaves	F: Boiled	2 L
*Ceratonia siliqua* L. Fabaceae (cultivated)	*Cascënieddë* G *Sciuscelle* G	Fruits	F: Consumed dried	9 G
			M: Dried and decocted for cold	4 G
*Chondrilla juncea* L. Asteraceae (wild) CALARB15	*Cotël* G	Young leaves	F: Raw, as salad and boiled	4 G
*Cichorium intybus* L. Asteraceae (wild)	*Cëcorië* GL	Young leaves	F: Raw, as salad and boiled M: Decoction, as a diuretic	40 G 48 L
		Young central stems	F: Raw, in salads	3 G
*Citrus × sinensis* (L.) Osbeck Rutaceae (cultivated)	*Arancë* G *Portuhallë* L	Peel	M: Decoction for cold	8 G 3 L
*Clematis vitalba* L. Ranunculaceae (wild) CALARB19	***Sciasciamiedd***** G** *Talvosë* **L** ***Vërosë***** GL** *Vitacchië* L	Sprouts	F: Boiled or fried in a frittata	30 G 26 L
*Clinopodium nepeta* (L.) Kuntze Lamiaceae (wild) *	*Menta* G	Leaves	F: Raw or cooked as a condiment	1 G
*Conium maculatum* L. Apiaceae (wild)	*Cëcuta* GL *Cëchitonë* L	Leaves	M: Decoction as a somniferous for donkeys and horses	1 G 1 L
*Sorbus domestica* (L.) Spach Rosaceae (semi-domesticated)	*Sorbë* GL	Fruits	M: Decoction for cold and anti-diarrhoea	4 G 2 L
*Cornus mas* L. Cornaceae (wild)	*Curnalë* GL	Fruits	M: Raw as fruit	2 G 19 L
*Crataegus monogyna* Jacq. Rosaceae (wild)	*Biancospinë* **LG** ***Bisciulinë*** **G** *Cerasolë* L *Cirasiell* GL *Scescëlin* G *Spinapodëcë* L	Fruits	F: consumed raw as a snack	6 G 12 L
			M: Decoction for cold, heart diseases, and for improving blood circulation	1 G 1 L
*Crepis vesicaria* L., *C. mollis*, and possibly other *Crepis* spp. Asteraceae (wild) UNISGLU024 UNISGLU022	*Cëcorië* GL	Young leaves	F: Boiled	7 G 3 L
*Cydonia oblonga* Mill. Rosaceae	*Melë Cutugnë* G *Perë Cutugnë* G	Fruits	M: Decoction for cold	2 G
*Cynara cardunculus* L. Asteraceae (wild) CALARB34	*Scalerë* L	Flower bud	F: Boiled	1 L
*Cynara scolymus* L. Asteraceae (cultivated)	*Carcioffël* GL	Leaves	M: Decoction for liver diseases	2 G 2 L
*Cynodon dactylon* (L.) Pers. Poaceae (wild)	*Gramegna* GL *Gramignizzë* G	Rhizome	M: Decoction for stomachache, flu and bladder	22 G 20 L
*Daucus carota* L. Apiaceae (wild)	*Carota* GL *Rarëc* GL	Aerial parts	F: Boiled	1 G
*Diplotaxis tenuifolia* (L.) DC. Brassicaceae (wild) UNISGLU006	*Ruca Selvatica* L	Leaves	F: Raw as salad	3 L
*Dipsacus laciniatus* L. Caprifoliaceae (wild)	*Cecalupo* G *Cardognë* L	Morning dew found between the stem and the leaves	M: to wash face and eyes	1 G
			M: topically for hair loss	1 L
*Dryopteris filix-mas* (L.) Schott Dryopteridaceae (wild)	*Filëcë* L	Root	M: Decoction for fever and infections	1 L
*Equisetum arvense* L. Equisetaceae (wild)	*Coda Cavallina* G	Stem	M: Decoction for stomachache and kidney problems	1 G
			F: Boiled or fried in a frittata	1 G
*Euphorbia helioscopia* L. Euphorbiaceae (wild)	*Tutumaglë* G	Latex from the stem	M: Latex applied to the tooth for tooth decay	1 G
*Fagus sylvatica* L. Fagaceae (wild)		Bark	M: Decoction for the belly and astringency	1 G
*Ficus carica* L. Moraceae (semi-domesticated) CALARB05	*Ficë* G L	Infructescence	M: Sun-dried and decocted for cold	8 G 9 L
		Latex from the infructescence	M: Applied to the pendulous fibroma skin	7 G
			M: Applied to a bee sting	3 L
*Foeniculum vulgare* Mill. Apiaceae (wild) CALARB11	*Fënucchë* GL *Fënucchiett* GL	Young leaves	F: Boiled	44 G 32 L
		Fruits	F: Dried, as a condiment in home-made sausages and biscuits	36 G 32 L
			M: Dried and decocted for stomachache	5 G 2 L
*Fragaria vesca* L. Rosaceae (wild)	*Frahulë* GL *Murieddë* L	Fruits	F: Raw as fruit	17 G 20 L
*Gladiolus italicus* Mill. Iridaceae (wild) *	*Unspecified*	Flowers	F: Raw sucked as a candy	1 G
*Glycyrrhiza glabra* L. Fabaceae (wild)	*Gurrizia* L	Root	M: Raw or decocted for cough	3 L
*Helianthus tuberosus* L. Asteraceae (cultivated)	*Taratufolo* G	Tuber	F: Raw or cooked as potatoes	1 G
*Helminthotheca echioides* (L.) Holub Asteraceae (wild) *	*Scëpredda* G *Sprignë* G *Sproiënë* L *Spruggën* G *Spruscët* L	Young leaves	F: Boiled	14 G 12 L
		Leaves	M: Raw rubbed on the eyes for chalazion	1 L
*Helosciadium nodiflorum* (L.) W.D.J.Koch Apiaceae (wild) UNISGLU001	*Accëtiedd* L *Scavunë* L	Leaves	F: Raw or cooked	8 L
*Hordeum vulgare* L. Poaceae (cultivated)	*Orzë* G L *Huorscë* GL	Fruits	F: Roasted and infused in water as coffee	6 G 1 L
			M: Decoction for cold	1 L
*Humulus lupulus* L. Cannabaceae (wild)	*Iuppët* G	Shoot	F: Boiled or fried in a frittata	3 G
*Hylotelephium spectabile* (Boreau) H.Ohba Crassulaceae (wild)	*Erba Della Madonna* G *Ghrassodda* L	Leaves	M: Raw applied to the skin on boils and pimples	1 G 10 L
*Hypochaeris radicata* L. Asteraceae (wild) *	*Mussë Purcieddë* L	Young leaves	F: Boiled	1 L
*Juglans regia* L. Juglandaceae (semi-domesticated)	*Cuzzunë* L *Nocë* GL *Vërdunë* G	Fresh husk	M: Boiled and placed on the skin for burns	2 L
			M: Infused in olive oil, mixed with ash for wounds	2 G
			M: Crushed, pressed, and filtered to obtain the juice, which is applied to the skin for wounds	1 G
		Kernel	M: Dried and decocted, used for cold	4 G 5 L
*Lactuca sativa* L. Asteraceae (cultivated)	*Lattuga* L	Leaves	M: Boiled and posed on the skin to counteract abscesses	1 G
*Lactuca serriola* L. Asteraceae (wild) *	*Scarola* G	Young Leaves	F: Boiled	1 G
*Laurus nobilis* L. Lauraceae (wild) CALARB10	*Laurë* GL	Leaves	F: Seasoning M: Decoction for cold and cough	9 G 5 L
		Fruits	M: Macerated in olive oil, used externally	1 G
*Leontodon hispidus* L. Asteraceae (wild) CALARB26	*Cicoria* GL	Young leaves	F: Boiled	1 G 1 L
*Leopoldia comosa* (L.) Parl. Asparagaceae (wild) *	*Civuddinë* GL	Bulbs	F: Soaked in water, boiled, and fried	22 G 19 L
*Lolium temulentum* L. Poaceae (wild)	*Giuoglë* G	Seeds	M: Crushed with flour for hallucinations and calming effects in humans and animals	1 G
*Malus domestica* Baumg. Rosaceae (cultivated)	*Melë* GL	Fruits	M: Decoction for stomachache	10 G 7 L
*Malva sylvestris* L. Malvaceae (wild) CALARB01	*Malvë* GL *Maulë* L	Leaves and flowers	M: Decoction for cold and stomachache	39 G 46 L
			M: Boiled, and the leaves were placed on the skin for wounds	1 L
			M: Decocted and placed on the abscess for a tooth infection	1 G
*Marrubium vulgare* L. Lamiaceae (wild)	*Marruggia* GL *Marruoghë* L	Leaves	M: Decoction for belly and liver problems	4 G 8 L
			M: Crushed, decocted, macerated in water overnight on burns	2 L
			M: Decocted and placed on the abscess for a toothache	1 G 1 L
			M: Decoction rubbed on the cow’s udder for mastitis	2 G
			M: Decocted and drunk as a vermifuge	1 L
			M: Fried in olive oil; the oil is applied to the skin for rashes	1 L
			M: Decoction applied to the cow’s hoof	1 G
			M: Decoction; the patient needs to sit above the smoke for haemorrhoids	1 G
*Matricaria chamomilla* L. Asteraceae (wild)	*Cambomill* GL *Hammamidë* GL	Leaves and flowers	M: Infusion for cold and stomachache	40 G 42 L
*Mentha longifolia* (L.) L. Lamiaceae (wild) *	*Mentë* GL	Leaves	F: Raw or cooked as a condiment	2 G 8 L
*Mentha pulegium* L. Lamiaceae (wild) *	*Mëndastrë* G *Puliescë* L	Leaves	F: Raw or cooked as a condiment	1 L
*Nasturtium officinale* R.Br. Brassicaceae (wild)	*Ravela* G *Masturzë* L	Leaves	F: Raw as salad	3 G 1 L
*Onopordum illyricum* L. Asteraceae (wild) *	*Cardo* L	Leaves	F: Boiled	1 L
*Origanum vulgare* L. Lamiaceae (wild) UNISGLU027	*Areganë* GL *Collëna* L	Leaves and flowers	F: Dried as a condiment	21 G 27 L
			F: Dried, decocted	1 L
*Orobanche crenata* Forssk. Orobanchaceae (wild)	*Favalupo* L *Neha* L	Shoots	F: Boiled or fried in a frittata	3 L
*Papaver rhoeas* L. Papaveraceae (wild) UNISGLU017	*Papaverë* GL *PapaverIna* L *Paparina* L	Whorls	F: Boiled	14 G 16 L
		Fruits	M: Decoction used as a somniferous for children	4 G 1 L
			M: Wrapped in a napkin, placed in the mouth for tooth pain	1 G
*Papaver somniferum* L. Papaveraceae (cultivated) UNISGLU018	*Papagna* GL *Papagnuolë* GL	Fruits	M: Decoction used as a somniferous for children	24 G 20 L
			M: Raw used for tooth pain	1 G
*Parietaria judaica* L. Urticaceae (wild) UNISGLU008	*Erba Rë Vindë* G	Stems and leaves	M: Decoction for kidney stones	3 G 4 L
*Pastinaca sativa* L. Apiaceae (wild)	*Bastianaccë* G *Radëghë* G	Root	F: Boiled	1 G
*Petroselinum crispum* (Mill.) Fuss Apiaceae (cultivated)	*Pëtrësin* G *Radëghë* G	Root	F: Boiled for Christmas	1 G
*Picris hieracioides* L. Asteraceae (wild) *	*Scëpredda* G *Cicoria* L	Young leaves	F: Boiled	1 G 2 L
*Pimpinella anisoides* L. Apiaceae (wild) UNISGLU003 UNISGLU004	*Anisetto* G *Ciminiedda* G *Ciuminë* G *Fënucchiettë Anice* G	Seed	F: Dried, seasoned home-made biscuits	21 G 16 L
*Plantago lanceolata* L. Plantaginaceae (wild)	*Orecchie Di Pecora* G	Leaves	F: Boiled	1 G
*Plantago major* L. Plantaginaceae (wild) UNISGLU002	*Cient Ervë* L G *Cientë Nervë* L	Leaves	M: Raw as a poultice on an abscess	1 G 3 L
*Portulaca oleracea* L. Portulacaceae (wild)	*Përchiazzë* L *Përchiachieddë* L *Përdiacca* G *Purchiacca* GL	Stems and leaves	F: Raw as salads	20 G 17 L
			M: Decoction for stomachache	1 G
*Prunus amygdalus* Batsch Rosaceae (semi-domesticated) UNISGLU034	*Mennël* G L	Seed	M: Dried and decocted for cold	9 G 4 L
		Exocarp	M: Decoction for cold	4 G 2 L
*Prunus cerasus* L. Rosaceae (wild)	*Amarena* L	Fruit	F: Raw as fruit	2 L
*Prunus domestica* L. Rosaceae (cultivated) UNISGLU033	*Auleggën;* *Ghuleggën* *Bulescën* G L	Fruits	M: Dried and decocted for cold	1 G 2 L
			F: Raw as fruit	11 G 10 L
*Prunus spinosa* L. Rosaceae (wild)	***Brignuolë*** **G** *Trignë* L ***Prumelle*** **G** *Prugnuol* L *Atregnël* L	Fruits	F: Overripe	20 G 22 L
			M: Decoction for belly pain and blood pressure	1 G
		Seed	M: Decoction used as a laxative	1 L
*Pteridium aquilinum* (L.) Kuhn Dennstaedtiaceae (wild)	*Filëcë*	Leaves	M: Raw used on skin swellings	1 G
*Punica granatum* L. Lythraceae (semi-domesticated)	*Granata*	Esocarp	M: Decoction for stomachache	3 G 3 L
*Pyrus communis* L. Rosaceae (cultivated) CALARB06	*Perë*	Pome	M: Decoction for cold	2 G 3 L
*Pyrus spinosa* Forssk. (wild)	*Perëpërainë* G *Peraggën* G *Përazz* G	Pome	M: Decoction for cold	5 G 1 L
*Reichardia picroides* (L.) Roth Asteraceae (wild) UNISGLU005	*Lengua Di Pecora* **GL**	Leaves	F: Boiled	1 G 2 L
*Robinia pseudoacacia* L. Fabaceae (wild)	*Caggia* GL	Flowers	F: Fried in frittata	4 G
			F: Raw sucked as candies	3 L
*Rosa canina* L. Rosaceae (wild)	***Ghratta Culë*** GL *Rosa Dë Li Ciuccë* G *Ruvascëcë* L	Pseudofruits	F: Raw as fruit and jams	9 G 7 L
			M: Decoction for flu and diarrhoea	6 G
*Rubus idaeus* L. Rosaceae (wild)	***Frammosa*** **G** *Brëgnuolë* GL	Fruit	F: Raw fruit	5 G 1 L
*Rubus ulmifolius* Schott Rosaceae (wild)	*Ra Morë* L *Ruëuitë* L *Rughularë* G *Ruvëtalë* GL	Fruit	F: Raw fruit	19 G 26 L
			M: Decoction for cold	1 G
		Leaves	M: Raw rubbed on the skin for wounds	5 G 5 L
			M: Heated over a fire for an abscess	2 G 2 L
			M: Posed on the eyes for a chalazion	2 L
*Rumex acetosa* L. Polygonaceae (wild) *	*Ervëacitë* GL *Garëgarucë* L	Leaves	F: Raw as salad	1 G 7 L
*Rumex crispus* L. and possibly other *Rumex* spp. Polygonaceae (wild)	*Ervëamarë* L	Young leaves	F: Boiled	2 L
*Ruscus aculeatus* L. Asparagaceae (wild)	*Pungitopo* GL *Vëruscimë* L	Shoots	F: Boiled or fried in a frittata	3 G 5 L
*Ruta chalepensis* L. (wild), Rutaceae UNISGLU009 and R. graveolens (cultivated) Rutaceae	*Ruta* GL	Aerial parts	M: Fried in olive oil for skin rashes	2 L
			M: Infused in water and drunk as a vermifuge	5 G
			M: Minced and mixed with *Allium sativum*, rubbed on the skin for bone pains	2 G 1 L
			M: Raw as a vermifuge for animals	3 G
			M: Crushed to obtain the juice, rest overnight and drink as vermifuge	2 G
			M: Infused in alcohol for digestion	1 L
			F: Raw as salad seasoning	1 G
			M: Raw as a poultice for skin swellings	1 G
*Salix alba* L. Salicaceae (wild)	*Salëcë* GL	Branches	M: Macerated in water for tooth pain	1 G 1 L
*Salvia officinalis* L. Lamiaceae (cultivated)	*Salvia* GL	Leaves	M: Decoction and drank for stomachache	3 G 2 L
			M: Decoction for poultice on skin swellings	3 G
*Salvia rosmarinus* Spenn. Lamiaceae (cultivated)	*RosaMarina* GL	Leaves	M: Decoction and drank for a cold	7 G 2 L
			M: Decoction for poultice on skin swellings	3 G 3 L
*Sambucus nigra* L. Adoxaceae (wild)	*Sammucë* GL *Savuccë* L *Scuppëtunë* GL	Flowers	M: Dried and decocted for cold and cough	27 G 23 L
			F: Breaded and fried	4 G 5 L
			M: Raw crushed as a poultice on wounds	2 L
			F: Infused in water as a syrup	1 L
		Fruits	F: Cooked for jams	2 G 3 L
			F: Crushed and drunk as juice	1 G
			M: Decocton for cold	1 G
		Bark	M: Decoction used on burns	2 G
		Leaves	M: Raw as a poultice on skin swellings	1 G
*Scolymus hispanicus* L. Asteraceae (wild) *	*Cardognë* L *Cardonë* G *Stiglione* GL	Mydribs	F: Boiled and fried	13 G 15 L
*Silene vulgaris* (Moench) Garke Caryophyllaceae (wild) CALARB32	*Erva Cuccë* L *Fronna Dë Cucëmariedd* L	Young leaves	F: Raw as salad and boiled	3 L
		Leaves	M: Heated over a fire and burned on the skin for burns	1 L
*Silybum marianum* (L.) Gaertn. Asteraceae (wild)	*Cardo Di San Giovanni* L *Cardunë* GL	Leaves	F: Boiled	4 G 7 L
*Sinapis arvensis* L. and possibly *Hirschfeldia incana* (L.) Lagr. -Foss. and Rapistrum rugosum (L.) All. (wild)	*Asënë* GL *Camammarelle* G *Erba Dë Campagna* **G** *Rëassëna* GL *Rapastrë Assëna* L *Rapazzë* G *Sënapa* L *Senapieddë* L	Young Leaves	F: Boiled	20 G 22 L
*Smilax aspera* L. Smilaceae (wild)	*Unnamed*	Root	M: Rubbed on the back for a backache	1 G
*Solanum chenopodioides* Lam. Solanaceae (wild) UNISLU021	*Unnamed*	Fruit	M: Posed on the tooth pain	1 L
*Solanum tuberosum* L. Solanaceae (cultivated)	*Patatë* GL	Skin of the tuber	M: Raw as a poultice for burns	1 G 4 L
		Tuber	M: Boiled and posed on the skin for huffy	2 G 2 L
*Sonchus oleraceus* L. Asteraceae (wild) CALARB14 and possibly other *S.* spp. (wild)	*Sëvun* G L	Young leaves	F: Boiled	45 G 40 L
*Spartium junceum* L. Fabaceae (wild)	*Ginestrë* GL *Scinestrë* GL	Stems	M: Medicinal decoction for fever	1 G
*Taraxacum officinale* F.H.*Wigg* and possibly other *T.* spp. Asteraceae (wild) UNISGLU010 and possibly other *T*. spp.	*Cicoria* L *Cëcurionë* L *Maroglia* GL *Marognë* G *Pisciacanë* **G** *Tarassacë* **G**	Young leaves	F: Raw as salad or boiled	22 G 14 L
			M: Raw used as a diuretic	1 G
*Thymus serpyllum* L. Lamiaceae (wild) *	*Serapoddë* G *Timo* L	Leaves	F: Dried as a condiment	1 L
			M: Decoction for cold	1 G
*Tilia cordata* Mill. Malvaceae (semi-domesticated)	*Tiglë*	Shoots and flowers	M: Decoction for cold	2 G
*Triticum turgidum* L. subsp. *durum* (Desf.) Husn. Poaceae (cultivated)	*Ghranë*	Husk	M: Decoction used to wash babies	2 G 1 L
			M: Roasted, applied to the skin for muscular pain	1 L
*Tussilago farfara* L. Asteraceae (wild)	*Cuoppë* G *Cuppëtieddë* L *Ognë Cavaddë* G	Leaves	M: Heated over a fire, topically applied to the abscess on the skin	3 G 3 L
			M: Roasted over a fire, posed on the skin for burns	4 G
			M: Boiled for stomachache	1 L
			M: Raw applied to the skin for snake bites	1 G
			M: Decoction for burning feet	1 G
*Ulmus minor* (Mill.) Ulmaceae (wild)	*Olmë* G	Samarae	M: Raw used for abscess	1 G
*Urospermum picroides* (L.) Scop. ex F.W.Schmidt Asteraceae (wild) *	*Cicoria* GL *Gallinella Grassa* **G** *Maroglia* L	Young leaves	F: Boiled	8 G 2 L
*Urtica dioica* L. Urticaceae (wild) CALARB04	*Ardicë* GL	Young leaves	M: Decoction	7 G 12 L
			F: Boiled for meatballs, pasta and soups	7 G 11 L
			M: Boiled for hair loss	4 G 3 L
			M: Raw, applied to the skin for rheumatism	1 G 3 L
		Root	M: Decoction for cold	1 L
*Valerianella* sp. Caprifoliaceae (wild)	*Valerianë* G	Leaves	F: Raw as salad or boiled	2 G
*Verbascum densiflorum* Bertol. Scrophulariaceae (wild)	*Mëndonia* L	Leaves	M: Decoction, the patient needs to sit above the smoke for haemorrhoids	2 L
*Vitis vinifera* L. Vitaceae (cultivated)	*Virë* GL	Fruits	M: Crushed and boiled for a cough	6 G 2 L
			F: Boiled	4 G 2 L
			M: Fermented into wine for wounds	2 G
			M: Fermented into wine for goat mastitis	1 L
			M: Fermented into wine, then mixed with sulphur and salt as an animal vermifuge	1 G
		New shoots	F: Boiled	1 G
*Xanthium spinosum* L. Asteraceae (wild) UNISGLU007	*Sulëfizë* G	Root	M: Boiled as a poultice for rheumatism	1 G
*Zea mays* L. Poaceae	*Mazzuoccël* GL	Silks	M: Decoction for cold	2 G 1 L
*Ziziphus jujuba* L. Rhamnaceae (wild) UNISLU010	*Scescëla* G	Fruits	M: Decoction for cold	13 G
Unidentified Asteraceae taxon (potentially *Lactuca* sp.) (wild)	*Cicurina* G	Leaves	F: Boiled	1 G
Unidentified Asteraceae (wild)	*Sëvonë Dëpretë* L	Young leaves	F: Boiled	2 L
Unidentified Asteraceae taxon (wild)	*Cicorie Dë Pretë* L	Leaves	F: Boiled	1 L
Unidentified Asteraceae taxon (wild)	*Culëscuertë* G	Leaves	F: Boiled	1 G
Unidentified taxon (wild)	*Petacchi* L	New shoots	F: Boiled	1 L
Unidentified taxon (wild)	*Piedi Teneri* L	Leaves	F: Boiled	1 L
Unidentified taxon (wild)	*Fronna Dë Pandonë* L	Leaves	M: Raw for wounds	1 L
Unidentified taxon (wild)	*Fronna Dë Cuccë* G	Leaves	M: Heated over fire for burns	1 G
Unidentified taxon (wild)	*Cuculë* G	Leaves	F: Battered and fried	1 G
Unidentified taxon (wild)	*Spaccacucchiara* L	Leaves	F: Boiled	1 L
Unidentified taxon (wild)	*Calcagnë Dë Cravë* G	Leaves	F: Boiled	1 G
Unidentified taxon (wild)	*Culanninë* L	Leaves	M: Dried and decoction as vermifuge	1 L

The quantitative indices computed on the contemporary dataset are summarised in Table 4 and Table 5. The 127 identified taxa yielded 2036 use reports, 1061 among Gallo-Lucanian and 988 among Lucanian participants when the emic taxa are included. Cultural importance is strongly concentrated: the mean CI across all taxa is 0.18 and the median only 0.06, while the ten most salient taxa account for 39% of all use reports. Eleven taxa reach RFC ≥ 0.50; that is, they were cited by at least half of the participants, whereas 64 taxa fall below RFC 0.05 and are held by a handful of individuals each. This distribution (a small, highly consensual core surrounded by a long tail of idiosyncratic knowledge) is the pattern typically reported for Mediterranean foraging repertoires, and it is the tail, not the core, that is most exposed to the demographic processes described below.

**Figure 4:**
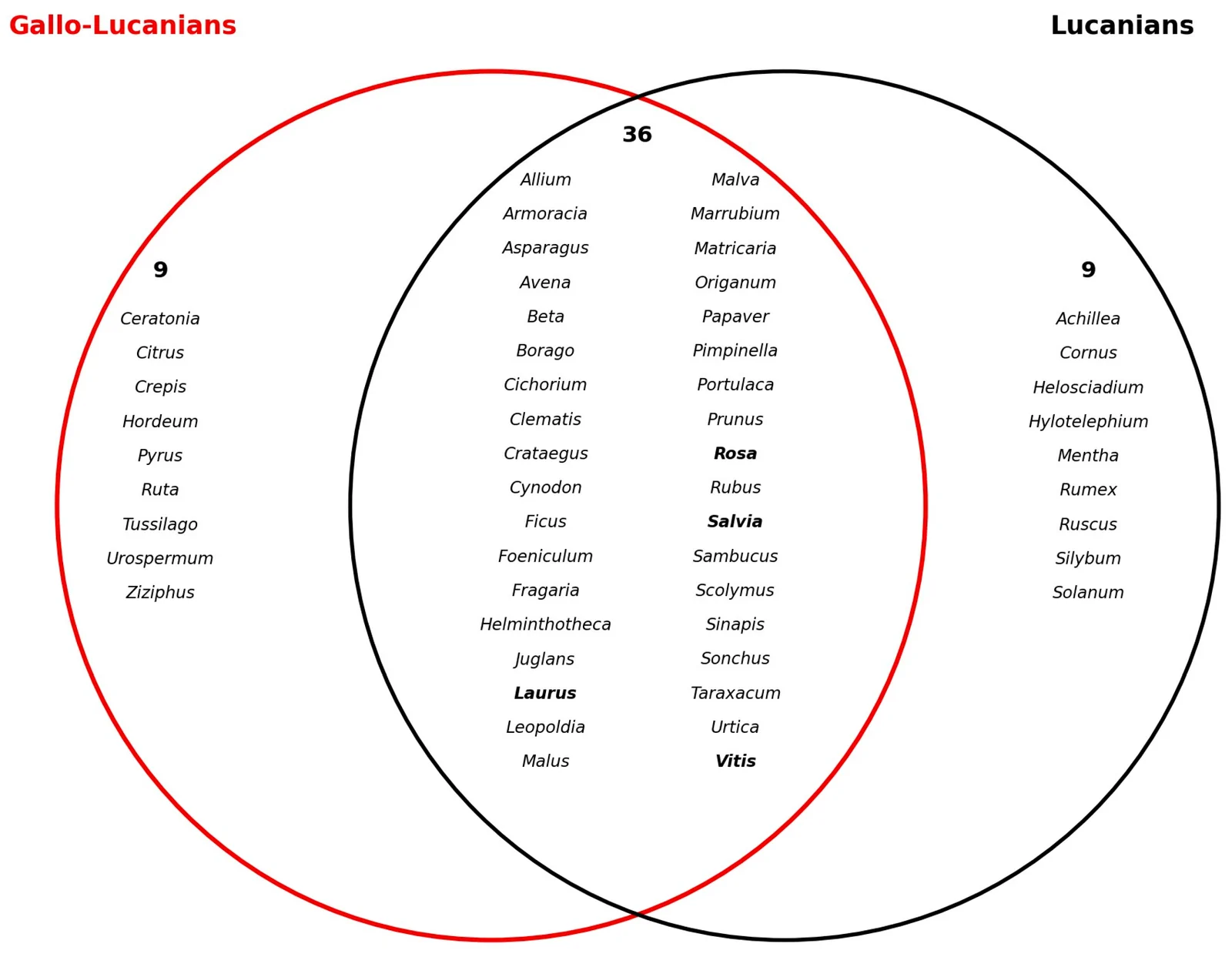
Cross-cultural comparison of the most frequently cited plant genera in the two communities. Counts are use citations, that is, the number of times a genus was reported for a given use, summed over all uses and over all participants of the community, as recorded in Table 3; because a participant reporting several uses of the same plant contributes more than one citation, they are not a count of individuals. Genera reaching at least five citations in a community are included. The Venn diagram shows the genera reaching that level in both communities (36) and those reaching it only among Gallo-Lucanians (9) or only among Lucanians (9). The four genera cited at least twice as often in one community as in the other are shown in bold.

*Foeniculum vulgare* is the culturally most important taxon in both communities (CI = 1.68 overall), followed by *Cichorium intybus*, *Malva sylvestris*, *Sonchus oleraceus* and Matricaria chamomilla. The ordering of the leading taxa is very similar in the two groups, which is what the Jaccard similarity of 0.58 and the largely shared genus set of Figure 4 would predict. Differences appear lower down the ranking and in relative emphasis rather than in the identity of the core taxa, and they are quantified by the chi-square test reported in Section 3.5.

Informant consensus is high across the board (Table 5). The wild food category reaches ICF = 0.94 and the medicinal category as a whole ICF = 0.91, values comparable to those reported for other inland Mediterranean communities and consistent with a well-defined, socially shared repertoire rather than with idiosyncratic individual practice. Among the medicinal categories, consensus is highest for gynaecological and paediatric uses (0.94), gastro-intestinal and hepatic complaints (0.93) and urinary and renal complaints (0.92), each of which is dominated by a small number of taxa. It is lowest for oral and dental uses (0.30) and ophthalmic uses (0.33), where the few use reports recorded are spread across almost as many taxa; these two values rest on 11 and 4 use reports respectively and should not be over-interpreted. The high consensus for the largest categories indicates that the domestic pharmacopoeia described in Section 3.4 is a genuinely collective body of knowledge, which strengthens the case for regarding its erosion as a loss of shared cultural capital rather than of individual recollection.

### 3.2. Landscape Change and the Ecology of the Harvested Repertoire

**Figure 3:**
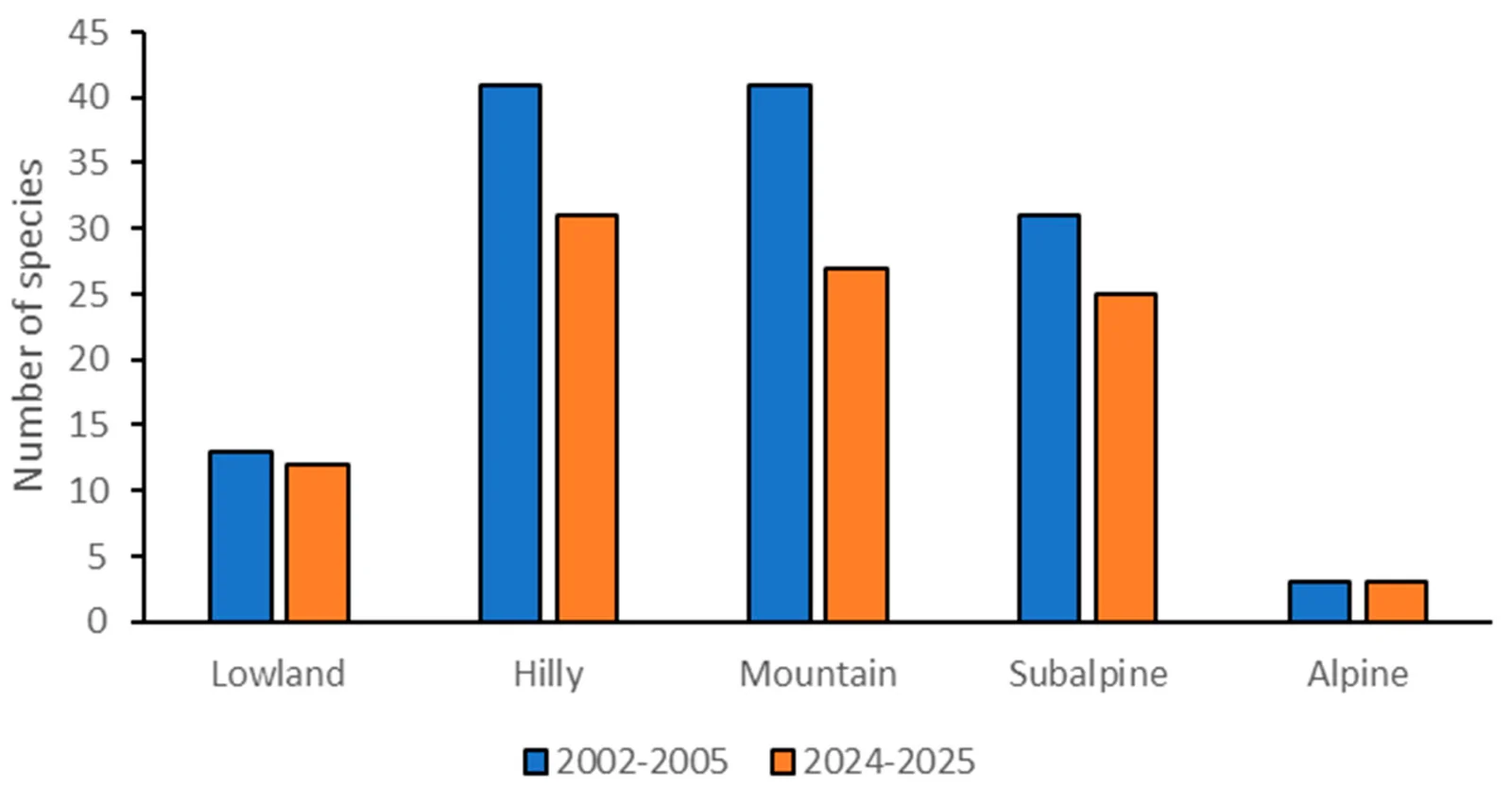
Number of harvested species grouped according to the highest elevational belt occupied by each taxon. Elevational belts are described in the Section 2

The ecological composition of the recorded taxa reflects historical interactions between human communities and disturbed Mediterranean landscapes. Comparing the taxa recorded in Basilicata in the 2024–2025 survey with those reported in the literature ([29,30]), the number of harvested plant species decreased from 129 to 110 (12 of the latter being emic taxa that could not be identified botanically). Considering the highest elevational belt reached by each species, the number of species whose range is confined to the lowland belt (0–200 m) remained relatively constant (12 and 13 species, respectively), whereas the numbers for the hilly, mountain and subalpine belts declined by 10, 14 and 6 species, and the number reaching the alpine belt remained constant (Figure 3). As noted in the Methods, these figures describe the potential elevational range of the taxa concerned and not the elevations at which they were gathered, so they indicate a contraction of the repertoire towards taxa of lower-elevation distribution rather than a measured retreat of gathering from high ground.

The observed decline in harvested taxa should not be interpreted merely as a consequence of species loss or reduced availability. Rather, it reflects the progressive disruption of everyday land-based practices through which ecological knowledge is generated and reproduced. Similar dynamics were recently documented in Mediterranean coastal and island communities, where land abandonment, migration, and tourism transformed ethnobotanical repertoires into fragmented cultural memory despite the continued availability of plants [22]. In Basilicata, pastoral decline appears to have produced a comparable decoupling between people and landscape, reducing opportunities for repeated ecological learning.

Accordingly, a comparison of wild plants gathered by Lucanians from 2003–2005 to 2024–2025 shows a noticeable decrease in the number harvested (127 and 87 genera, respectively), with synanthropic taxa dominant in both periods. This decline is consistent with, and may partly reflect, the abandonment of agropastoral systems in the Basilicata Region, which began in the 1950s and is especially evident in sheep numbers, which fell from about 40,000 to 15,000 head between 2007 and 2016 [40,41]. Differences in survey design between the two datasets nevertheless prevent causal attribution, and part of the difference may be methodological rather than temporal.

The abandonment of pastures characterised by herbaceous cover was followed by reforestation through natural secondary succession, a trajectory documented across much of Mediterranean Europe wherever low-intensity agro-pastoral systems have been withdrawn [42]. Considering the FAO LCCS1 Land Cover Classification, from 2001 to 2019 more than 110,000 hectares of dense herbaceous cover were lost, while an increment of more than 80,000 hectares of sparse forest (tree cover 10–30% with canopy > 2 m) and 26,000 hectares of deciduous broadleaf forest was registered [43]. Despite this, only three of the newly recorded plants were trees (*Juglans*, *Punica* and *Salix* spp.), suggesting that while landscape change may reduce the number of harvested plants, the composition of the repertoire remains shaped by the ecological knowledge of those who gather in the same habitats. The decrease in grassland’s extent produces a more homogeneous landscape, favouring ecological connectivity for forest-associated species such as wild ungulates and wolves, while reducing habitat suitability for ecotonal and open-meadow species of conservation interest. This raises concerns about human–wildlife conflict ([44] and references therein), especially since large carnivores and ungulates preferentially move along roads, trails, and open corridors that also give gatherers access to the landscape [45]. Whether their presence deters gathering was not investigated here: no participant was asked about wildlife encounters, and the possibility is raised as a hypothesis for future work rather than as a documented driver of ethnobotanical change.

Pasture abandonment is not the only driver of change. In the Basilicata Region, as in most of Italy, long-term multi-year drought events were detected in 2006–2008, 2011–2013, 2017–2018 and 2021–2023 [46], and drought has also increased fire frequency over time, contributing to changes in vegetation [47]. The decline in harvested plants is therefore consistent with pasture abandonment [48] acting alongside broader landscape change, although neither factor was tested directly against the ethnobotanical data. Despite this decline, the number of harvested plants remains higher than in the adjacent regions of Calabria and Campania [49,50]. One possible contributing factor, which the present data cannot test, is the emergence of so-called “community-based tourism” in the region, a form of tourism managed by local communities that valorises their resources and culture [51]. Although Basilicata remains an underexploited tourist region, visitor numbers rose from 944,108 to 1,035,462 between 2019 and 2025 [52], and exports of Lucanian culinary products rose by 62% between 2019 and 2021 ([51] and references therein). It is therefore conceivable, though not demonstrated by our dataset, that the erosion of ethnobotanical knowledge associated with pasture abandonment has been partially offset by growing interest in local food products [51,53]. Comparable heritagisation processes, in which wild plants are sold as traditional local products in restaurants or offered as eco-tourism experiences, are emerging across Europe [54,55]. If so, gathering pressure may increase as heritagisation advances [15]. We emphasise that no harvesting-pressure or population-level abundance data were collected in this study: overexploitation is raised as a potential conservation concern for the region, not as an observed phenomenon, and monitoring of the most commercially salient taxa, in collaboration between civil society and academics, would be required to establish whether it is occurring [56].

Synanthropic and ruderal species constitute a substantial proportion of the documented ethnobotanical dataset, confirming the importance of anthropogenic habitats and human activities in shaping local ethnobotanical repertoires. Species associated with field margins, roadsides, cereal fields, abandoned terraces, and pastoral corridors were particularly prominent.

This ecological pattern is historically coherent with the agro-pastoral economy that characterised inland Basilicata until recent decades. Traditional systems of transhumance, mixed farming, low-intensity cereal cultivation, and domestic animal husbandry generated highly heterogeneous ecological mosaics favourable to ruderal edible species and medicinal herbs. The ethnobotanical repertoire therefore reflects not wilderness-oriented gathering practices, but rather intimate interaction with human-shaped landscapes.

Woodland and forest-edge taxa also occupied an important position, particularly among Gallo-Lucanian communities. Species such as *Allium ursinum*, *Rubus idaeus*, *Fagus sylvatica*, *Castanea sativa*, and Equisetum arvense point to familiarity with cooler, more humid ecological niches often associated with northern mountain environments. This is consistent with the selective maintenance of ecological preferences linked to ancestral settlement histories, although an equally plausible reading is the simple ecological availability of these taxa in the moister sites where several Gallo-Lucanian villages are located; the present design cannot distinguish the two. A recent analysis of hourly rainfall from 2001 to 2024 indicates a stronger increase in precipitation at higher altitudes in the study area [57], which may have favoured these species, but we did not test this association.

Overall, the results indicate that ethnobotanical knowledge in the study area should be understood as a biocultural system historically produced through continuous interaction among linguistic identity, agro-pastoral subsistence, ecological disturbance, and local landscape management. The observed transformations in plant use patterns therefore reflect broader processes of socio-economic modernisation, rural abandonment, and linguistic erosion that simultaneously affect both ecological knowledge and cultural memory.

### 3.3. Wild Food Plants and the Importance of Synanthropic Species

Wild food plants represented the dominant functional category within the recorded ethnobotanical corpus. The overwhelming prevalence of boiled leafy vegetables, mainly consumed blanched and pan-fried or in teas, reflects the persistence of a Mediterranean, subsistence-oriented culinary system historically associated with peasant and pastoral economies in inland Southern Italy, and places the Lucanian repertoire squarely within the wider circum-Mediterranean tradition of gathered greens described for Italy, Spain, Greece, Cyprus and the Levant [58,59]. Very commonly quoted taxa such as *Cichorium intybus* (40 G; 48 L), *Foeniculum vulgare* (44 G; 32 L), *Sonchus oleraceus* (39 G; 36 L) and Papaver rhoeas (14 G; 16 L) remain culturally salient across communities and generations.

The ecological distribution of these species is particularly significant. Most belong to synanthropic, ruderal, and agro-pastoral habitats strongly associated with anthropogenic disturbance regimes. Their abundance in ethnobotanical memory reflects the historical centrality of mixed agro-silvo-pastoral systems characterised by cereal cultivation, seasonal grazing, open pathways, field margins, and low-intensity agricultural practices. Consequently, the persistence of these species in local cuisine may reflect ecological continuity linked to traditional land-use systems.

However, the data also indicate processes of ecological restructuring. Several taxa associated with transhumant landscapes and northern mountain ecologies appear today as residual or memory-based practices rather than active traditions. Genera such as *Allium*, *Humulus* and *Valerianella* spp. survive primarily among older participants and show low citation frequencies despite their ethnobotanical prominence in northern Italy. This pattern is compatible with agricultural intensification, the abandonment of traditional pastoralism, rural depopulation and reduced interaction with forest-edge habitats, though these processes were not measured in the present study.

The frequent citation of highly bitter wild greens, including *Cichorium*, *Clematis*, *Helminthotheca*, *Hirschfeldia*, *Rapistrum*, *Sinapis*, *Sonchus*, *Taraxacum*, *and Urospermum* spp., is equally important from a cultural perspective. Bitterness is a recurrent sensory element in Mediterranean peasant food systems and often carries symbolic associations with purification, seasonal transition, and bodily regulation. The maintenance of these taste preferences among elderly informants suggests that food memory remains strongly tied to ecological familiarity and historical experiences of scarcity.

### 3.4. Medicinal Knowledge and Domestic Healthcare Systems

Medicinal applications were among the most diverse components of the dataset. Treatments addressed gastrointestinal disorders, respiratory infections, kidney ailments, skin conditions, fever, rheumatism, vermifuge practices, and veterinary care. The medicinal repertoire reveals a domestic healthcare system deeply integrated with everyday subsistence activities and ecological accessibility.

Frequently cited medicinal taxa included *Matricaria chamomilla*, *Malva sylvestris*, *Sambucus nigra*, *Cynodon dactylon*, *Marrubium vulgare*, *Ruta graveolens*, *and Laurus nobilis*. Many preparations involved decoctions, infusions, poultices, fumigations, and macerations in olive oil or wine, demonstrating the persistence of highly localised pharmacological traditions rooted in household practice rather than formalised herbal medicine.

Several remedies reveal strong overlap with ethnobotanical traditions documented in northern Italy. The medicinal uses of *Ruta graveolens*, *Foeniculum vulgare*, *Papaver rhoeas*, *Taraxacum officinale*, *Achillea millefolium*, *and Rosa canina* correspond closely with uses previously reported in Piedmont and Liguria. Such continuities reinforce the hypothesis that medicinal knowledge may preserve long-term cultural stratifications associated with migration history.

*Ruta graveolens* deserves particular comment, since it is among the most frequently cited medicinal taxa in both communities and is used here, as elsewhere in inland Southern Italy, in macerations and decoctions for digestive and menstrual complaints and as an anthelmintic. A recent critical review of the species brings together its traditional uses across Eurasia, its phytochemistry—dominated by furanocoumarins, quinoline alkaloids, essential oils, and flavonoids—and experimental evidence for its anti-inflammatory, antimicrobial, and cytotoxic activities [60]. That review also documents the toxicological profile of the species, including its phototoxic furanocoumarin content and its abortifacient potential, which is directly relevant to the local practices recorded here: the emmenagogue and abortifacient uses reported by our participants correspond to the pharmacological activity described in the literature, and the same constituents account for the dermatitis associated with handling the fresh plant in sunlight. The persistence of these uses in Lucania is therefore not merely a cultural survival but a practice with a documented pharmacological and toxicological basis, and one whose safety implications should be made explicit whenever such traditions are valorised commercially.

At the same time, the medicinal repertoire also displays considerable adaptation to local Southern Italian ecological conditions. Species strongly associated with Mediterranean xeric environments, including *Ruta chalepensis*, *Spartium junceum*, *and Origanum vulgare*, occupy a prominent role in Lucanian domestic medicine. This combination of inherited northern traditions and adaptive incorporation of southern taxa demonstrates that ethnobotanical systems are dynamic rather than static cultural survivals.

### 3.5. Cross-Cultural Comparison

Figure 4 shows the cross-cultural comparison of the most frequently cited plant genera, based on the use citations recorded in Table 3 and restricted to genera reaching at least five citations in a community. Thirty-six genera reach that level in both communities, nine only among Gallo-Lucanians and nine only among Lucanians. Across the full dataset, 74 of the 127 identified taxa were cited by both communities, 30 only by Gallo-Lucanians, and 23 only by Lucanians, giving a Jaccard similarity of 0.58 between the two repertoires. The communities nevertheless differ in the relative weight given to individual taxa: a chi-square test on the taxon × community matrix of citation counts, restricted to the 65 taxa with at least five citations, rejects independence (χ^2^ = 132.0, df = 64, *p* < 0.001). The pattern is thus one of a largely shared repertoire cited with significantly different emphasis. This is consistent with a long history of intermarriage and exchange between the Gallo-Lucanian settlers and the autochthonous Lucanians, although the present design cannot separate this mechanism from differences in the ecological settings of the respective villages.

The reviewers rightly ask whether the differences between the two groups can be attributed to cultural inheritance once ecological availability is taken into account. We can address this only partly, and we state the limits plainly. The two sets of villages lie in the same phytogeographical district, on the same southern Apennine substrate, within a narrow elevational band (568–954 m a.s.l. for the Lucanian municipalities, 650–954 m for the Gallo-Lucanian ones), and no taxon recorded in one group of villages is absent from the flora of the other; the ecological setting is therefore approximately, though not exactly, held constant by design. We did not collect the site-level data—georeferenced gathering locations, local abundance, habitat availability per municipality—that would allow ecological opportunity to be entered as a covariate and its contribution formally partitioned from that of language group. The chi-square result therefore establishes that the two repertoires are cited with significantly different emphasis; it does not establish that the difference is cultural in origin. Where the two explanations can be told apart at all in our material, it is in the lexicon rather than in the flora, as Section 3.6 sets out: the northern element survives as a set of names attached to plants that both communities gather in comparable quantities, which is difficult to explain ecologically but straightforward to explain by household transmission.

### 3.6. Vernacular Phytonyms and the Erosion of a Minority Lexicon

The vernacular nomenclature documented in the study is one of the dataset’s most significant dimensions. Phytonyms preserve evidence of linguistic contact, historical migration, ecological adaptation, and cultural memory. The coexistence of Gallo-Lucanian and Lucanian lexical forms for the same taxa demonstrates the layered nature of local linguistic ecologies. It reflects centuries of interaction between northern settler communities and Southern Italian populations.

A few vernacular names show affinities with northern Italian forms and appear to be absent from the surrounding Lucanian dialects. Seven of the folk names recorded, marked in bold in Table 3 and referring to five taxa, have no traceable counterpart in the Southern Italian dialectological literature and are formally compatible with attested Piedmontese or Ligurian forms; names that are merely absent from the immediate area, but pan-Italian in form, were not marked. These forms may mark a Gallo-Lucanian ethnobotanical heritage. Establishing this, however, would require systematic comparison with documented Piedmontese and Ligurian phytonyms, identification of regular phonological correspondences, and consultation of historical linguistic sources, none of which was undertaken here; the proposed northern affinities are therefore preliminary and would need a dedicated ethnolinguistic study to confirm. All of these phytonyms refer to plants used by both communities.

This can be examined more precisely by cross-tabulating the phytonyms of northern ancestry against the citation data. The seven names marked in bold in Table 3 attach to five taxa: *Clematis vitalba* (*Sciasciamiedd*, *Vërosë*), *Crataegus monogyna* (*Bisciulinë*), *Prunus spinosa* (*Brignuolë*, *Prumelle*, compare Piedmontese *Brignola*), *Rosa canina* (*Ghratta culë*, compare Piedmontese and Lombard *Gratacü*) and Rubus idaeus (*Frammosa*, compare *Frambosa*). All five are cited above the threshold by both communities, and with closely comparable frequencies: *Clematis* (30 Gallo-Lucanian citations versus 26 Lucanian), *Prunus* (46 versus 43), *Rubus* (32 versus 36) and Crataegus (7 versus 13). None is confined to either community. The single exception is *Rosa* (15 versus 7), which one community cites at least twice as often as the other.

Those four genera are worth a moment’s attention, because they run in a single direction: *Vitis* (14 Gallo-Lucanian citations versus 5 Lucanian), *Salvia* (16 versus 7), *Rosa* (15 versus 7) and Laurus (10 versus 5) are all cited more heavily by the Gallo-Lucanians, and no genus in the shared set shows a difference in comparable size in the opposite direction. All four are domestic plants of the house and the garden rather than the open countryside—the vine, sage, the dog rose of the hedgerow, the bay tree beside the door—and Rosa is the only one carrying a phytonym of northern form. The pattern is suggestive rather than conclusive, since it rests on four genera and on citation counts that are not counts of individuals. Still, it points to the domestic sphere as the setting in which whatever distinctiveness remains is most likely to be found, which is also where the surviving lexicon is transmitted.

The implication is uncomfortable but, we think, important. The northern lexical inheritance is not attached to a distinct botanical repertoire: it survives on plants that both communities gather, in comparable quantities, for the same purposes. There is no Gallo-Lucanian flora in inland Lucania, and on the evidence assembled here there has not been one for a long time. What nine centuries of coexistence have left is a handful of words, and the practices those words once described have converged completely with those of the surrounding Lucanian population.

This is the expected outcome, and it should be stated as such rather than presented as a disappointment. Gathering practice is constrained by what grows within walking distance of the village, and the settlers moved into a flora that had nothing in common with the one they left; adaptation to the local vegetation was a condition of survival, not a cultural choice, and it would have been substantially complete within a few generations. Vocabulary, by contrast, is transmitted within the household and carries no ecological cost, so it persists far longer than the practices it names. The erosion of the Gallo-Lucanian ethnobotanical heritage in Lucania is therefore effectively total at the level of practice and residual at the level of language. The residue is more tenuous still than the word “residual” suggests: as noted in Section 2.2, there is no discrete Gallo-Lucanian variety left to erode, only a substratum audible in intonation, prosody, a characteristic sing-song cadence and a scattering of individual lexical items, of which the phytonyms discussed here are among the most durable. What the phytonyms preserve is not an alternative way of using plants but the memory of a migration, and that is the last stage before a biocultural inheritance ceases to be recoverable at all.

A further observation reinforces the point. Several of these phytonyms were produced by participants who could no longer describe how the plant was prepared, or who had not gathered it for decades: in these cases, the word has outlived the practice it named.

This last pattern bears directly on how biocultural erosion should be modelled. It is often assumed that language and ecological knowledge decline together, and that losing a minority variety entails losing the ecological knowledge encoded in it. Our material suggests a more sequential process: gathering practice ceases first, detailed procedural knowledge follows, and the name persists longest, surviving as a lexical residue in the speech of the oldest generation. If this ordering is general, then phytonym inventories in minority communities function as a leading indicator of what has already been lost in practice, and as a lagging indicator of what is about to be lost in language. It also implies that the interval between the two—the number of plants still named but no longer used—is itself a measurable quantity, and one that future surveys in minority contexts could usefully record.

The practical consequence is that documenting a minority lexicon becomes urgent precisely when its ecological content has already begun to disappear. In the Gallo-Lucanian villages, the participants who still produced these forms were, with few exceptions, over sixty, and we encountered no younger speaker who used them spontaneously. Because what survives is a set of individual items embedded in an otherwise Lucanian dialect rather than a system transmitted as a whole, these forms are unusually exposed: an isolated lexeme can drop out of use without leaving any structural gap behind it, and nothing in the surrounding dialect requires its replacement. On present trends, the phytonyms discussed here will pass out of active use within one generation, taking with them the last accessible trace of a nine-century-old migration. We stress, at the same time, that the northern attribution of these forms remains preliminary: establishing it securely would require systematic comparison with documented Piedmontese and Ligurian phytonyms, identification of regular phonological correspondences, and consultation of historical dialectological sources, none of which we undertook here. A dedicated ethnolinguistic study, conducted jointly by ethnobiologists and dialectologists, is the obvious next step and, given the demographic situation, a matter of some urgency.

The persistence of vernacular names for plants that are no longer commonly used is especially important. In several cases, informants retained highly specific phytonyms even when detailed knowledge regarding preparation or collection had partially eroded. This suggests that language may preserve fragments of ethnobotanical memory beyond the active survival of practices themselves. Consequently, linguistic persistence may outlast ecological or practical continuity.

The data further demonstrate that ethnobotanical knowledge transmission occurs through multilingual and culturally hybridised processes rather than through isolated linguistic systems. Numerous taxa possess parallel Gallo-Lucanian and Lucanian names used interchangeably depending on context, generation, or social interaction. This phenomenon complicates simplistic narratives of cultural isolation and instead points towards long-term processes of coexistence, borrowing, and ecological co-adaptation.

These findings directly address broader debates concerning the relationship between minority languages and traditional ecological knowledge. The present study demonstrates that linguistic erosion does not merely entail vocabulary loss but may also contribute to the disappearance of ecological classifications, gathering practices, seasonal knowledge, and culturally embedded perceptions of the landscape. The erosion of minority languages and dialects therefore entails a parallel reduction in biocultural diversity.

### 3.7. Diachronic and Cross-Cultural Analysis

**Figure 5:**
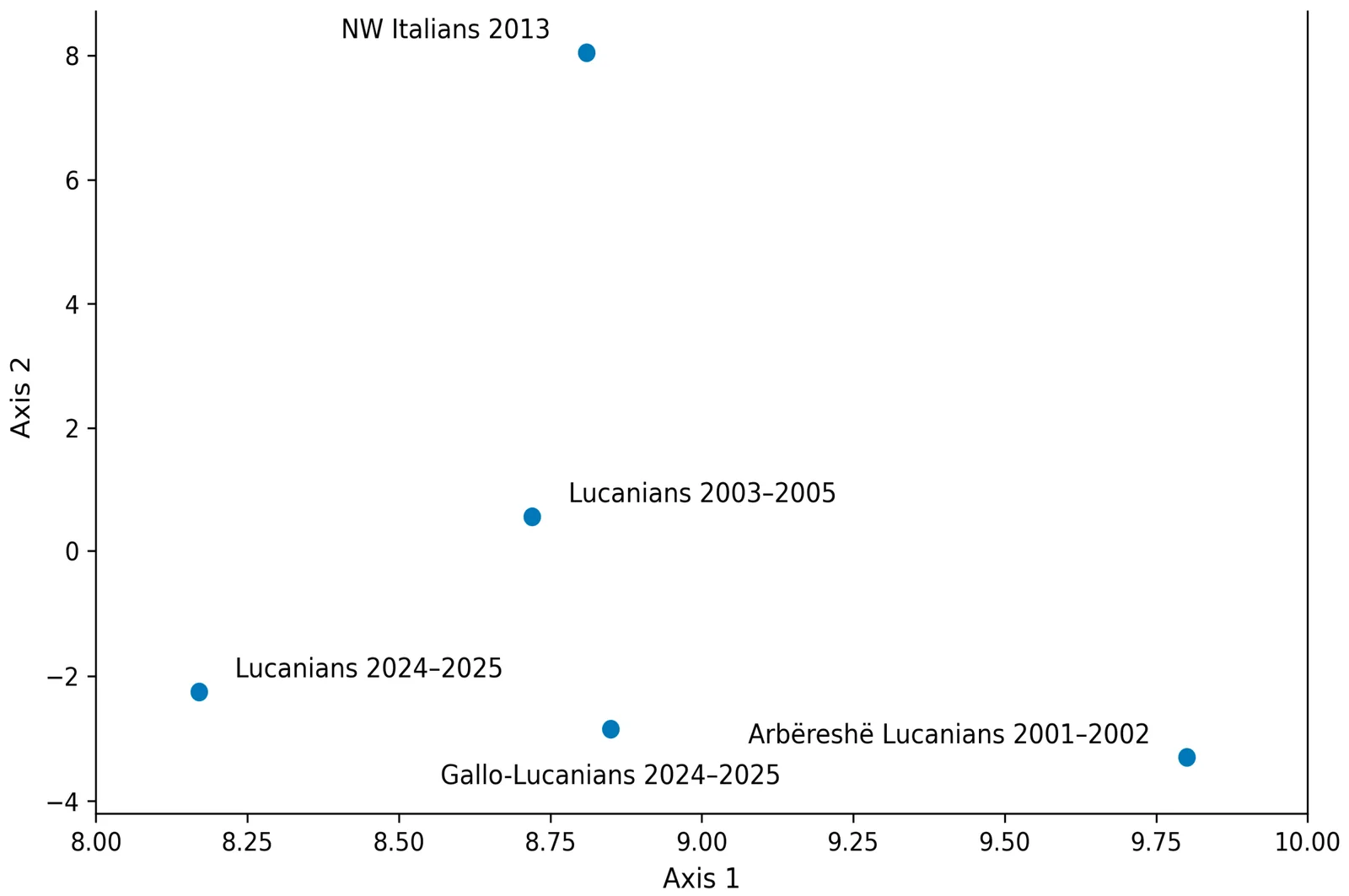
Detrended Correspondence Analysis (DCA) ordination of five ethnobotanical repertoires from inland Lucania and North-Western Italy, computed on a presence/absence matrix of 225 plant genera. Repertoires (with the number of genera recorded in each): Gallo-Lucanian 2024–2025 (95), Lucanian 2024–2025 (86), Arbëreshë of Lucania 2000–2001 (110), Lucanian 2002–2003 (122) and North-Western Italian 2009–2011 (144). Genera used only as cultivated food plants were excluded, because the food surveys among the sources recorded non-cultivated plants only; medicinal uses were retained whether the plant is gathered or grown. Cells record the presence of the genus, not citation frequency; sources are listed in Table 2, and the matrix is provided as Supplementary Material.

Detrended Correspondence Analysis (DCA) showed differentiation among the ethnobotanical datasets along both temporal and cultural dimensions (Figure 5). The north-western Italian dataset occupied a distinct position along Axis 2, while the Gallo-Lucanian communities were positioned between the north-western Italian and Lucanian datasets. The contemporary Lucanian dataset (2024–2025) showed partial separation from the historical Lucanian dataset (2003–2005), and the Arbëreshë communities occupied a relatively isolated position along Axis 1.

Before reading the pattern, it is worth restating precisely what was compared, since this determines what the figure can and cannot show. Five repertoires were ordinated—Gallo-Lucanian 2024–2025, Lucanian 2024–2025, Arbëreshë of Lucania 2000–2001, Lucanian 2002–2003 and North-Western Italian 2009–2011—on the presence or absence of 225 plant genera (Section 2.4). The unit is the genus, the matrix excludes genera used only as cultivated food, and the datum is presence, not frequency: two communities that gather the same genus appear identical on that genus whether it is a staple or a single recollection. Figure 5 therefore maps the composition of the five repertoires against one another, and the axes should be read as gradients of shared and unshared genera rather than of ethnobotanical importance.

The four datasets entering the ordination differ considerably in origin and in size, and this must be borne in mind when reading Figure 5. The contemporary Gallo-Lucanian and Lucanian datasets each derive from 45 interviews conducted by the same researcher in 2024–2025 with a common interview guide. We assembled each historical dataset by merging two earlier studies by our group in Basilicata: the Arbëreshë dataset combines the survey of weedy vegetables in the Vulture area [28] with the ethnopharmacy of the Arbëreshë of northern Basilicata [32]. In contrast, the Lucanian dataset combines the culinary uses of non-cultivated vegetables and mushrooms recorded in a southern Italian village [29] with the comparative survey of traditional pharmacopoeias among Albanians and Italians of southern Italy [30]. The north-western Italian dataset comes from the survey of the North-Western Ligurian Alps carried out in 2013 by a team of the University of Genoa and published the following year [31]. For these merged and external datasets, the number of participants, the recruitment procedure, and the range of use categories recorded were not reported in a form that allows direct comparison with the present survey, and merging two sources per historical dataset further increases their heterogeneity. The datasets therefore differ in sampling intensity, and some of the separation along both axes may reflect this heterogeneity rather than differences in ethnobotanical knowledge. We report the ordination as an exploratory description of the material and refrain from inferring from the distances between dataset centroids.

The ordination describes the structure of the datasets and does not by itself identify the processes generating it. The intermediate position of the Gallo-Lucanian assemblage is consistent with long-term cultural persistence combined with adaptation to Southern Italian agro-pastoral systems; the separation of the two Lucanian datasets is consistent with a diachronic restructuring of local ecological knowledge over the last two decades, plausibly linked to declining pastoral practice and reduced interaction with marginal habitats; and the relatively isolated position of the Arbëreshë is consistent with differentiated ethnobotanical trajectories associated with linguistic and cultural distinctiveness. Each of these readings is a hypothesis consistent with the ordination rather than a result of it, and differences in sampling design, use categories and recording intensity among the datasets compared may themselves contribute to the observed separation.

Overall, the ordination is consistent with ethnobotanical repertoires in Southern Italy being shaped by historical migration processes, ecological exposure, and ongoing socio-economic transformation rather than by geographic proximity alone. Still, the design of the comparison does not allow estimation of the relative contribution of these factors.

### 3.8. Folk Plant Uses, Crop Wild Relatives and the Mediterranean Inner Areas

The documentation of folk plant uses is sometimes regarded as an antiquarian exercise, valuable for the record but marginal to contemporary concerns. The case examined here suggests otherwise, and it is worth setting out why this kind of botany matters beyond its own discipline.

The first reason is nutritional and agronomic. The wild greens that dominate the Lucanian repertoire—*Cichorium intybus*, *Sonchus oleraceus*, *Foeniculum vulgare*, *Borago officinalis*, *Papaver rhoeas* and their companions—are precisely the taxa now attracting attention as underused resources for diversified and resilient food systems ([37,61,62]). They grow without irrigation, without fertiliser and without cultivation, on soils that are marginal for conventional agriculture, and they are gathered in the seasons when cultivated vegetables are scarce. In a Mediterranean climate becoming hotter and more erratic, a repertoire of species already adapted to drought and disturbance is not a museum piece but an inventory of options, and the communities that still hold it are the only reliable source of information on how these plants are recognised, harvested at the right stage, and rendered palatable.

The second reason concerns the Mediterranean inner areas specifically. Inland Basilicata belongs to that band of mountainous, sparsely populated, and ageing territories that stretches across Southern Europe and that national policy in Italy has come to designate as *aree interne*: places distant from essential services, losing population for decades, and repeatedly identified as the weak link in territorial cohesion. These same areas hold a disproportionate share of the continent’s biocultural diversity, including most of its surviving linguistic minorities. The knowledge documented here is therefore concentrated exactly where demographic decline is sharpest, and any strategy to revitalise these territories that ignores it discards one of the few distinctive assets they retain.

That asset has a demonstrable economic dimension. The valorisation of local food products, foraging-centred gastronomy, and community-based tourism has already been shown to generate income in comparable Italian contexts ([15,51]), and the growth in visitor numbers and culinary exports reported above indicates that the mechanism is operating in Basilicata as well. Folk plant knowledge gives such initiatives their content: without it, a foraging menu or an eco-tourism itinerary is an invention rather than heritage. Conversely, as we noted earlier, commercial valorisation risks increasing harvesting pressure on the most marketable taxa, and the sustainability of any such development depends on monitoring that has not yet been established [56].

The third reason is environmental governance. Local ecological knowledge encodes long-term observations of habitats, phenology, and disturbance regimes that no monitoring programme can reconstruct retrospectively and integrating it into landscape management has repeatedly been shown to improve both biodiversity outcomes and the legitimacy of conservation measures among affected people ([5,21,63]). In a region where more than 110,000 hectares of herbaceous cover have converted to woodland within two decades, the gatherers interviewed here are among the few observers who can describe what that landscape contained before the transition, and what has disappeared with it.

A fourth reason, and for the study of plant genetic resources the most consequential, is that the repertoire documented here is unusually rich in crop wild relatives. At least fifteen of the taxa gathered in the study area belong to the primary or secondary gene pools of European crops: *Beta vulgaris* in its wild form, *Daucus carota*, *Pastinaca sativa*, *Lactuca serriola*, *Cynara cardunculus*, *Asparagus acutifolius*, *Diplotaxis tenuifolia*, *Chenopodium album*, *Rumex acetosa*, *Allium schoenoprasum*, *Foeniculum vulgare*, *Portulaca oleracea*, *Prunus spinosa*, *Pyrus spinosa* and the *Sinapis arvensis* complex together with *Hirschfeldia incana* and Rapistrum rugosum. These are not incidental presences: several rank among the most frequently cited taxa in the dataset, and gatherers distinguish, name, and select among their populations on criteria of taste, texture, tenderness, and season that have no counterpart in formal descriptor lists.

The Mediterranean Basin is one of the world’s principal centres of crop wild relative diversity. For a specific historical reason: it is where much of the European vegetable, fruit and forage repertoire was domesticated, and where the wild progenitors of those crops have continued to coexist with their cultivated descendants for millennia within the same agricultural landscapes. Beet, carrot, parsnip, lettuce, artichoke, rocket, fennel, asparagus, plum and pear all have wild relatives that are native, widespread and still gathered around the basin. This coexistence distinguishes the Mediterranean from regions where crop wild relatives survive only in remnant natural vegetation: here they are, and always have been, plants of the cultivated countryside itself.

That characteristic is also the source of their vulnerability, because it means that crop wild relatives in the Mediterranean occupy exactly the ground that agricultural modernisation removes. They are plants of disturbed and transitional habitat, and they persist in the interstices of the farmed landscape rather than within protected areas: on terrace risers and dry-stone walls, along field margins, droveways and irrigation channels, on the edges of abandoned pastures, and in the ruderal ground around farmsteads and threshing floors. Herbicide use, field enlargement, the removal of margins and the sealing of tracks eliminate these habitats first, and formal conservation seldom targets them, since they lie on land that no inventory classifies as of conservation interest. Mediterranean crop wild relative populations are therefore among the least protected components of European plant genetic resources, despite being among the most valuable.

Against this background, the inner areas acquire a particular significance. In the mountains of Basilicata, cultivation has remained low-intensity, chemical inputs limited, field patterns small and terrace systems largely intact, and the abandonment of pasture has so far produced secondary succession rather than land conversion. The interstitial habitats that crop wild relatives require therefore survive here in quantity, and with them populations that have never been subject to the selective sweep imposed by intensive agriculture elsewhere in Europe. The inner areas of the Mediterranean function, without anyone having designed them to, as a diffuse and unregistered reserve of crop wild relative diversity, and their value in this respect is inversely proportional to their economic weight.

That reserve is not secure, and the threat is the opposite of what operates in the lowlands. The same succession that has preserved these habitats until now is progressively closing them: the land-cover figures reported above record the conversion of more than 110,000 hectares of herbaceous cover to sparse and deciduous woodland within two decades, and the light-demanding ruderal and grassland species that supply most of the crop wild relative pool are the first to be shaded out. Recurrent drought and increasing fire frequency add further pressure, and their effects on these populations are unknown. Inner-area crop wild relatives are thus caught between intensification in the valleys and canopy closure on the slopes, and conventional monitoring is unlikely to detect their decline because nobody is looking at the ground they occupy.

This is where documenting folk plant uses acquires an applied value that goes well beyond the ethnographic record. Gatherers know where these populations are, how they behave from year to year, which stands have disappeared and when, and which sites reliably produce tender growth in a dry spring. That knowledge is spatially precise, temporally deep and, for the taxa concerned, unavailable from any other source; it constitutes a practical starting point for the in situ inventory of crop wild relatives in territories that national genetic resource programmes have largely passed over. It also distinguishes populations under active use pressure from those being lost to neglect, which bears directly on the choice between in situ management and ex situ collection. The selection criteria applied by gatherers deserve particular attention, since traits such as reduced bitterness, delayed bolting, tenderness after boiling or persistence through drought correspond to characters of direct interest in breeding, and the populations repeatedly chosen for these qualities constitute a pre-screened sample that no random collecting strategy would reproduce. Coupling ethnobotanical survey with targeted collecting in interstitial habitats, before the generation able to locate them is gone, would convert a record of cultural memory into an addition to the European plant genetic resource base [64].

None of these argues for a defensive or nostalgic conservation of tradition. It argues that folk plant knowledge should be treated as an operational resource for the sustainable development of marginal Mediterranean territories—for food diversification, for rural income, for landscape management—and that its documentation is urgent for the same reason that the knowledge is valuable: in the inner areas, the generation that holds it is the last one that will.

## 4. Limitations of the Study

Several limitations should be borne in mind when interpreting the results presented above.

First, participants were recruited through snowball sampling and opportunistic encounters, with a preference for individuals locally recognised as knowledgeable and for elderly farmers, shepherds and gatherers. The sample is therefore not representative of the general population of the study area, and the repertoire described here should be read as the knowledge held by the most knowledgeable segment of these communities rather than as an average level of ethnobotanical competence.

Second, voucher coverage is incomplete, and this is the study’s principal taxonomic limitation. Of the 127 identified taxa, 36 carry an herbarium code, but 15 of these refer to specimens collected during earlier surveys in Basilicata and Calabria: only 21 vouchers, of which 15 correspond to wild taxa, were collected during the present fieldwork. The remaining taxa were identified in the field or in photographs. For the majority, this is unproblematic, since they are common trees, shrubs, ferns and unmistakable herbs, or cultivated species. For sixteen taxa, marked with an asterisk in Table 3, no plant specimen or photograph was available, and the identification rests on the vernacular name and on the description given by the participant; most of these belong to genera that are difficult to determine from description alone, in particular the rosette-forming Asteraceae gathered as wild greens and the genera *Mentha*, *Thymus*, *Allium* and *Rumex*. Because several of these taxa contribute to the discussion of bitter wild greens and of northern floristic affinities, the corresponding interpretations should be regarded as provisional until vouchered material becomes available. A targeted collecting campaign in the following gathering season, focused on these taxa, would resolve the issue; alternatively, they could be reported at genus level, which would preserve the ethnolinguistic evidence while removing the taxonomic uncertainty.

The reason for the incomplete voucher coverage should be stated, since it is remediable. Specimens were collected during fieldwork more often than the herbarium now reflects. Some material was damaged or developed mould during transport and storage and could not be accessed; therefore, for several taxa, the surviving documentation is photographic rather than physical. The first author holds the photographic record, which is available on request. A targeted collecting campaign in the next gathering season, using improved field-pressing and transport procedures, would close this gap for the taxa concerned.

Third, the comparison with historical datasets rests on material collected by different teams, with different sampling strategies and different recording intensities, and the metadata needed to assess comparability are only partly available, as set out in Section 3.7. Differences between periods may therefore reflect methodological differences as well as genuine change in local ecological knowledge, and the diachronic results are presented as indicative rather than as measurements.

Fourth, the elevational analysis is based on the potential upper range of each taxon rather than on the elevation at which it was actually gathered, since gathering locations were not systematically georeferenced. It describes the floristic composition of the repertoire and not the spatial behaviour of gatherers.

Fifth, the socio-ecological processes invoked in the discussion–pasture abandonment, drought, wildlife presence, tourism and culinary commercialisation–were not measured against the ethnobotanical data. They are drawn from independent regional sources and are offered as plausible interpretations, not as tested drivers.

Sixth, the linguistic interpretation of vernacular phytonyms is preliminary. A rigorous demonstration of Gallo-Lucanian ancestry for these forms would require systematic comparison with documented Piedmontese and Ligurian phytonyms, establishing regular phonological correspondences, and consulting historical dialectological sources.

The nature of the material compounds this limitation. There is no discrete Gallo-Lucanian variety against which the phytonyms could be checked internally: the Gallo-Lucanian element survives as a substratum within a Lucanian dialect, expressed in prosody, intonation and isolated lexical items rather than as a system. Establishing the northern ancestry of a given form therefore depends entirely on external comparison with documented Piedmontese and Ligurian material, which we have not undertaken. Our assignment of the seven bold names in Table 3 should be read accordingly, and the linguistic atlas of Basilicata [24] provides the obvious starting point for the dialectological work required.

Finally, we collected no data on harvesting pressure or the population status of the gathered species. Hence, the sustainability considerations raised above reflect a potential concern rather than a documented one.

An eighth limitation concerns participant reporting and the quantitative indices. We recorded gender, approximate age, principal occupation, educational level, length of residence, and length of gathering experience, but we did not retain individual ages; therefore, we can report only group means, and the sample cannot be described at the level of detail a stratified design would permit. Both subsamples contain more men than women, which may bias the recorded repertoire towards taxa associated with pastoral and field work relative to those associated with domestic food preparation. The same design feature—a use-centred rather than participant-centred recording unit—means no participant × taxon matrix exists, so the RFC values in Table 4 are lower bounds rather than measurements. At the same time, CI, UV, and ICF, defined on use reports, are unaffected. The ninth limitation is that the two communities were not matched on ecological availability at the site level, so the significant difference in citation emphasis reported in Section 3.5 cannot be partitioned between cultural inheritance and local ecological opportunity.

Future work in the region would benefit from a larger, more systematically stratified sample; georeferenced gathering data; a dedicated ethnolinguistic analysis of the phytonyms; and repeated surveys using a stable protocol, which would allow genuine longitudinal comparison in place of the retrospective comparison attempted here.

## 5. Conclusions

The present study demonstrates that the ethnobotanical systems of the Gallo-Lucanian and Lucanian communities of Basilicata constitute dynamic biocultural assemblages shaped through centuries of interaction among migration history, linguistic persistence, ecological adaptation, and agro-pastoral subsistence. Rather than representing static survivals of traditional knowledge, the documented plant repertoires reveal ongoing processes of cultural selection, ecological restructuring, and socio-economic transformation.

The analysis highlights the persistence of a broad ethnobotanical substratum shared across both linguistic groups, particularly regarding wild food plants, domestic medicinal practices, and synanthropic taxa associated with traditional rural landscapes. At the same time, the study identifies culturally specific botanical elements that maintain strong affinities with northern Italian ethnobotanical traditions documented in Piedmont and Liguria. The survival of these taxa, vernacular phytonyms, and associated practices is consistent with the long-term resilience of migration-derived biocultural knowledge systems; it does not, however, prove uninterrupted transmission over nine centuries, since later contact between northern and southern Italy could also explain part of the observed pattern.

The results further indicate that ethnobotanical knowledge persists through active ecological engagement rather than through memory alone. Species linked to traditional pastoralism, woodland gathering and low-intensity agriculture remain embedded in local knowledge primarily where direct interaction with these habitats continues. In contrast, the progressive abandonment of agro-pastoral practices, rural depopulation and the decline of subsistence economies coincide with the selective erosion and reorganisation of ethnobotanical repertoires. Knowledge associated with marginal habitats, seasonal gathering and vernacular medicinal systems appears particularly vulnerable. Among the factors reported to contribute to pasture abandonment is the lower productivity of autochthonous sheep and goats, which yield around 50% less milk and 30% less meat than commercial breeds [65]; the same authors argue that, with adequate rural policies, sheep and goat biodiversity can recover and livestock farms can be recognised as adding value to their territories.

The dataset’s linguistic dimension also shows that minority languages function as reservoirs of ecological classification and cultural memory. Vernacular phytonyms preserve historical layers of migration, adaptation, and ecological familiarity that botanical inventories alone cannot fully capture. The erosion of minority linguistic systems therefore entails not merely lexical loss, but also the disappearance of culturally embedded ecological knowledge, landscape perception, and intergenerational modes of environmental transmission.

From a broader theoretical perspective, the study contributes to debates on biocultural diversity by showing how language, ecology, mobility, and socio-economic change interact in producing and maintaining local ecological knowledge. The findings support approaches that interpret ethnobotanical systems as historically contingent socio-ecological processes rather than isolated collections of plant uses. Safeguarding local ecological knowledge should therefore be part of broader sustainability strategies, since such knowledge contributes to food resilience, cultural continuity, and adaptive resource management [66]. Integrating local ecological knowledge into landscape governance can also improve biodiversity conservation and strengthen community-based stewardship of rural environments [63].

The present findings indicate that documentation alone is insufficient to safeguard biocultural heritage: without sustained socio-ecological interaction, plant knowledge risks surviving only as memory rather than as practice. Future research should further integrate diachronic ethnobotanical comparison, ecological modelling, historical linguistics, and land-use analysis to clarify the mechanisms underlying biocultural persistence and transformation in Mediterranean inland regions. Greater attention should also be paid to the relationships among minority languages, ecological memory and changing patterns of rural landscape management amid demographic decline and environmental change. Safeguarding local ecological knowledge therefore requires conservation strategies that address not only species and habitats, but also the cultural processes that sustain human–environment relations [67].

## Figures and Tables

**Table 1:** Sampling frame, selection criteria and recorded characteristics of the 90 participants interviewed in 2024–2025.

Item	Value
Fieldwork period	August 2024–July 2025 (11 months)
Municipalities	15 (5 Gallo-Lucanian-speaking, 10 Lucanian-speaking)
Participants	90 (45 Gallo-Lucanian, 45 Lucanian)
Mean age	70 years overall; 72 years among Gallo-Lucanian and 68 years among Lucanian participants; oldest participant 92 years
Gender	Gallo-Lucanian: 27 men, 18 women. Lucanian: 27 men, 18 women. Total: 54 men, 36 women
Education	Primary schooling; no participant reported secondary or tertiary education
Residence	All participants lifelong residents of their municipality (an inclusion criterion)
Inclusion criteria	Long-term residence in a study municipality; local recognition as a holder of traditional plant knowledge; advanced age, or, for younger participants, direct transmission from a grandparent
Exclusion criteria	Prolonged residence outside the area through migration, employment or education, judged to have substantially displaced locally transmitted knowledge
Recruitment	Opportunistic encounters in villages, on agricultural land, in pastures and in woodland, complemented by snowball sampling through local social networks
Interview settings	Field margins and gathering sites; village streets; participants’ homes
Principal occupation	Farmers, shepherds and pensioners (largely retired agricultural workers), together with a smaller number of participants involved in traditional food preparation; recorded as a broad category rather than as a coded occupational variable
Group assignment	Residence in a municipality documented as Gallo-Lucanian [10,24] or in one of the ten surrounding Lucanian-speaking municipalities; see text above
Years of gathering experience	Lifelong: all participants reported having gathered wild plants since childhood

**Table 4:** The twenty culturally most important taxa (ranked by CI) among the 90 participants. UR = use reports; RFC = relative frequency of citation, reported as a lower bound and capped at 1.00 for the reasons set out in Section 2.4; CI = Cultural Importance Index, computed for the pooled sample and separately for each community (N = 45 each).

Taxon	UR	RFC (Lower Bound)	CI	CI (Gallo-Lucanian)	CI (Lucanian)
*Foeniculum vulgare*	151	0.84	1.68	1.89	1.47
*Cichorium intybus*	91	0.98	1.01	0.96	1.07
*Malva sylvestris*	87	0.94	0.97	0.89	1.04
*Sonchus oleraceus*	85	0.94	0.94	1.00	0.89
*Matricaria chamomilla*	82	0.91	0.91	0.89	0.93
*Sambucus nigra*	72	0.56	0.80	0.84	0.76
*Rubus ulmifolius*	62	0.50	0.69	0.60	0.78
*Borago officinalis*	60	0.52	0.67	0.80	0.53
*Clematis vitalba*	56	0.62	0.62	0.67	0.58
*Origanum vulgare*	49	0.53	0.54	0.47	0.62
*Urtica dioica*	49	0.21	0.54	0.42	0.67
*Asparagus acutifolius*	47	0.52	0.52	0.53	0.51
*Papaver somniferum*	45	0.49	0.50	0.56	0.44
*Prunus spinosa*	44	0.47	0.49	0.47	0.51
*Cynodon dactylon*	42	0.47	0.47	0.49	0.44
*Sinapis arvensis*	42	0.47	0.47	0.44	0.49
*Leopoldia comosa*	41	0.46	0.46	0.49	0.42
*Portulaca oleracea*	38	0.41	0.42	0.47	0.38
*Fragaria vesca*	37	0.41	0.41	0.38	0.44
*Pimpinella anisoides*	37	0.41	0.41	0.47	0.36

**Table 5:** Informant Consensus Factor (ICF) by use category. Use reports spanning more than one ailment were assigned to the first ailment named by the participant. Categories based on fewer than 15 use reports are reported for completeness but are not interpreted.

Use Category	N_ur_ (Use Reports)	N_t_ (Taxa)	ICF
Gastro-intestinal and hepatic	286	20	0.93
Respiratory	171	22	0.88
Dermatological and wound care	116	26	0.78
Gynaecological and paediatric	87	6	0.94
Urinary and renal	60	6	0.92
Oral and dental	11	8	0.30
Cardiovascular and metabolic	7	3	0.67
Veterinary	6	3	0.60
Ophthalmic	4	3	0.33
Other and unspecified	45	5	0.91
All medicinal uses	793	76	0.91
Wild food uses	1252	75	0.94

## Data Availability

The original contributions presented in this study are included in the article/Supplementary Material. Further inquiries can be directed to the corresponding authors.

## Supplementary Materials

The following supporting information can be downloaded at https://www.mdpi.com/article/10.3390/plants15172731/s1. DCA matrix. The genus × dataset presence/absence matrix (225 genera, five repertoires) underlying the Detrended Correspondence Analysis of Figure 5 is provided as a separate spreadsheet file.
